# Effect of polyamine precursors and antioxidants on growth and metabolism of salt-stressed barley

**DOI:** 10.12688/f1000research.130979.1

**Published:** 2023-03-10

**Authors:** Eman Eldakkak, Mohamed El-Shourbagy

**Affiliations:** 1 Botany Department, Faculty of Science, Tanta University, Tanta, Gharbia Governorate, Egypt

**Keywords:** Salinity Stress, Antioxidants, Polyamines

## Abstract

**Background:** Salinity is a serious problem that reduces crop productivity by affecting seed germination and seedling growth. It affects plant growth by disrupting plant osmosis, causing ionic toxicity, and metabolic and physiological changes. The aim of the present study was to examine the effect of polyamine precursors, besides the effect of some antioxidants such as glutathione and ascorbate on growth, metabolism, and productivity of two barley cultivars, different in salt tolerance, when subjected to salt stress.

**Methods:** Salt-tolerant G124 and salt-sensitive G119 barley cultivars had their seeds planted in plastic pots with clay and sand soil that measured 10 cm in diameter and 4 cm in height (2:1). The pots were divided into five categories: the grains were presoaked for 24 hours in distilled water, an amino acid solution (Arginine, Methionine, and Ornithine) (1 mM each), glutathione, or ascorbic acid (0.1 mM for each), seedlings were cultivated in 0.1 mM sodium chloride alone, seedlings were cultivated using a combination of salt and glutathione, seedlings were grown using a combination of salt and ascorbic acid, seedlings were cultivated using a combination of salt and amino acid mixture.

**Results:** Salinity has resulted in a reduction in all growth parameters in both barley cultivars. In the seedling, pre-flowering, and yield stages, the exposure to salt decreased photosynthetic pigments, total soluble carbohydrates, weight, and the number of grains, whereas it showed an increase in the activities of catalase and peroxidase, proline content, malondialdehyde, and membrane leakage.

**Conclusion:** The addition of glutathione, ascorbic acid, or amino acid mixture alleviated the harmful effect of sodium chloride and improved barley's defense mechanism against salt stress. The results showed that the alleviating effect was eventually reflected in plant growth, indicating that glutathione might be suggested as an effective treatment to reduce the impacts of salinity on barley.

## Introduction

Salinity is one of the most prevalent abiotic pressures in arid or semi-arid settings ( Koca *et al.*, 2007). High salinity has a negative impact on plants at the whole-plant level, resulting in plant mortality or a drop in productivity. Many plants develop mechanisms either to exclude salt from their cells or to tolerate its presence. Because of the negative effect of salt stress on crop productivity, crop productivity loss might exceed 60% of crop yield globally. Salinity stress disrupts physiological processes in plant cells, inhibits plant growth, and causes significant damage to photosynthesis ( Yan *et al.*, 2018), resulting in declines in pla nt production quantity and quality ( Taize & Zeiger, 2006). Furthermore, salinity has impacted plant growth and development by increasing salt content, particularly ionic chloride cl and sodium Na+, leading to changes in water status, mineral uptake, stomatal behavior, carbon allocation, and ion imbalance, resulting in water and osmotic potential disturbance ( Hasanuzzaman *et al.*, 2013). Salt stress reduces stomatal conductance, transpiration rate, and intercellular CO _2_ concentrations by decreasing photosynthesis ( Khoshbakht *et al.*, 2018; Xu *et al.*, 2018). Plants respond to salt stress by producing biochemical compounds and activating molecular systems at the cellular and plant levels ( Li *et al.*, 2019). Most cultivated plant species are highly sensitive and either die or display reduced productivity after exposure to salt stress for long periods. Yancey *et al.* (1982) reported that salt stress induced physiological and biochemical changes in the whole plant so that various compounds are synthesized such as abscisic acid, organic acids, proline, and polyamines (PAs). Polyamines are phytohormones that can be utilized to protect plants from abiotic stresses and to induce flowering ( Liu *et al.*, 2015). Because PAs levels change significantly in response to environmental stress, it has been reported that they are part of a plant's stress defense mechanism ( Turano & Kramer, 1993). Polyamines are involved in various plant development processes, including cell division, embryogenesis, floral and reproductive organ development, fruit ripening, root growth, leaf senescence, and response to biotic and abiotic stressors such as salt stress ( Sarwat *et al.*, 2013). Zhao and Qin (2004) suggested that polyamines improve all morphological and physiological characteristics and prevent chlorophyll degradation while also increasing the accumulation of all organic compounds under salt stress. In recent years, attention has been focused on the possible roles of the conjugated forms of polyamines in plants exposed to unfavorable environmental conditions ( Bouchereau *et al.*, 1999), but even so, only a few reports on the response of bound polyamines to salinity stress factors are available.

It has become widely known that reactive oxygen species (ROS) are to responsible for a diverse variety of stress-induced damage to macromolecules and, ultimately, cellular structure ( Sairam *et al.*, 2002). As a result, the role of antioxidant enzymes such as superoxide dismutase (SOD), ascorbate peroxidase (APX), glutathione reductase (GR), and catalase (CAT), as well as metabolites such as ascorbic acid, glutathione, -tocopherol, flavonoids, and carotenoids (CAR), in the quenching of ROS becomes critical ( Soliman *et al.*, 2019). Non-enzymatic antioxidants (glutathione and ascorbate) accumulated in root tissues of plants treated to salt stress, according to Vaidyanathan *et al.* (2003). Glutathione (GSH) is the most abundant non-protein thiol found in animal, plant, and bacterial cells. It contributes to the structural integrity of cells and has the potential to be utilized in the redox control of cell division ( Hossain *et al.*, 2017). Glutathione's physiological importance in plants comes from its role in sulfur metabolism and defense, where it is a vital source of reduced sulfur ( Smirnoff & Wheeler, 2000). Many important plant functions are regulated by it, including photosynthesis, DNA biosynthesis and repair, protein biosynthesis, amino acid transport, and enzyme control and activation. GHS has recently been discovered to play an important role in protecting chickpea plants from drought stress by regulating the antioxidant defense system and osmolyte synthesis ( El-Beltagi *et al.*, 2020).

Ascorbic acid (AA), is one of the most important and most abundant growth promoters presents in plants ( Khan *et al.*, 2011). A small amount of AA produced endogenously is involved in the promotion of the development and growth of plant cells. Ascorbic acid is involved in the phytohormonal mediated signaling pathways and is bound towards a various number of environmental stress conditions along with development and growth ( Barth *et al.*, 2006).

In view of the lack of knowledge regarding the role of polyamines in salt tolerance of grain crops, it is attempted in the present study examines the effect of polyamine precursors; arginine, methionine, and ornithine, besides the effect of some antioxidants as glutathione and ascorbate on growth, metabolism, and productivity of two barley cultivars, different in salt tolerance, when subjected to salt stress.

## Methods

### Preliminary experiment

Grains of eight barley cultivars (Giza117, Giza119, Giza 121, Giza 123, Giza 124, Giza 125, Giza 126 and Giza 2000) obtained from the Egyptian Agricultural Research Center were tested for germination under different concentrations of NaCl (50 mM, 100 mM, 150 mM, and 200 mM). Giza 124 was chosen as the most tolerant cultivar and Giza 119 as the most sensitive one with 100 mM NaCl to be used in the present study.

### Pot experiment

Grains of two cultivars; salt-tolerant G124 and salt-sensitive G119 of barley ( *Hordeum vulgare* L.) were germinated in plastic pots (10 cm diameter × 4 cm height each), filled with clay sandy soil (2:1). The pots were divided into five groups:

1- Grains were presoaked for 24 hrs in distilled water or amino acid mixture (Arginine, methionine and ornithine) 1mM for each or 0.1 mM for glutathione or1 mM for ascorbic acid.
2- One-week old seedlings were grown with 0.1mM sodium chloride alone.
3- One-week old seedlings were grown under the combined effect of 0.1 mM glutathione plus salt.
4- One-week old seedlings were grown under the combined effect of 1mM ascorbic acid plus salt.
5- One-week old seedlings were grown under the combined effect of 1mM amino acid mixture plus salt.

### Analysis of plant material

**Growth criteria**

After 21 d of exposure to each treatment, plants were harvested and separated into shoots and roots then shoot height, root depth, and leaf area were determined and weighed directly for the fresh weight (FW). Plant parts were dried in an air-driven oven at 80°C until constant weight for 48 hrs to determine the dry weight (DW).

**Chlorophyll determination**

A sample of fresh leaves (0.1 g) was homogenized in 5 ml 85% cold acetone at the seedling and pre-flowering stages, then centrifuged for 15 minutes at 3000 rpm and the pigment extracts were stored overnight in a refrigerator. The acetone extract was diluted to the appropriate volume, and the color intensities were measured at 663, 644, and 452.5 nm with a spectrophotometer to determine chlorophyll a, chlorophyll b, and carotenoids ( Metzner *et al.*, 1965).

**Measurement of photosynthetic efficiency**

In the seedling and preflowering stages, photosynthetic efficiency (Fv/Fm) of dark-adapted leaves was measured with a portable pulse amplitude modulation (PAM) fluorometer (PerkinElmer, UK). Mature leaves, morphologically similar, were dark-adapted for 30 min then placed in the leaf clip, to maintain constant angles of incidence between the fibre-optic arm of the fluorometer and the leaf surface. The measurement of photosynthetic efficiency, on the adaxial leaf surface, was carried out as described by ( Gonçalves & Santos Júnior, 2005). The leaf was initially exposed to the weak modulated measuring beam (< 0.1 umol/m ^2^ per s), to estimate the initial fluorescence (F0), when the PS II reaction centers are open (oxidized). Thereafter, the leaf was then exposed to 800 ms saturation pulse of high-intensity (>10000 umol/m ^2^ per s white light), to produce a transient closure (reduction) of the PS II reaction centers, at which point the maximum fluorescence (Fm), the variable fluorescence (Fv = Fm – F0), the maximum photochemical efficiency of PSII (Fv/Fm) and time of achieving maximum fluorescence yield (Tm) were obtained. Three replicates of each treatment were measured.

**Determination of total soluble carbohydrates**

The phenol sulfuric acid method has been used to estimate total soluble carbohydrates in the seedling, preflowering, and yield stages according to ( Dubois *et al.*, 1956).

Borate buffer extraction of dried samples: One g of dry shoot or root was incubated with 5 ml borate buffer (pH8) for 24 h and then centrifuged for 15 min at 3000 rpm. The supernatant was completed to a known volume, then 0.1 ml of sample solution was transferred into a test tube and 1ml of phenol and 5 ml of sulfuric acid were added, then put in a water bath at 25 °C for 20 min. The color was read at 490 nm.

**Determination of proline**

The techniques according to Bates *et al.* (1973) and Zúñiga *et al.* (1989) were applied for the determination of proline in seedling and yield stages. One-half g of dry tissue was homogenized in 5 ml of 3% (w/v) sulfosalicylic acid and then filtered. To 2 ml of filtrate, 2 ml acid ninhydrin was added, followed by 2 ml glacial acetic acid then boiled for 60 min, and the reaction was terminated in an ice bath. The reaction mixture was extracted with toluene and mixed vigorously in a separating funnel for 20 sec. The chromophore containing toluene was aspirated from the aqueous phase and the absorbance was read at 520 nm, using toluene as a blank.

**Assaying of antioxidant enzymes**

Fresh leaves (0.5 g) of each barley cultivar were ground with 8 ml of 50 mM cold phosphate buffer (pH 7.8) and centrifuged at 4000 rpm for 20 min. The supernatant was used for the determination of the activities of antioxidant enzymes in the seedling stage.

Catalase (EC 1.11.1.6) was assayed ( Kato & Shimizu, 1987) by measuring the initial rate of disappearance of H _2_O _2_. A sample of 3 ml of reaction mixture containing 0.1 M sodium phosphate buffer of pH 7.2, 11.8 mM H _2_O _2,_ and 0.1 ml enzyme extract. The decrease in H _2_O _2_ was followed by a decline in the absorbance at 240 nm and the activity was calculated using the extinction coefficient (40 mM ^-1^ cm ^-1^ at 240 nm) for H _2_O _2_. The activity was expressed in units of μM of substrate converted per minute per one gram of fresh weight.

The activity of peroxidase (EC 1.11.1.7) was assessed using ( Kato and Shimizu, 1987). The final assay volume was 3 ml, and the assay medium contained 0.1 M sodium phosphate buffer, pH 5.8, 7.2 mM guaiacol, 11.8 mM H _2_O _2_, and 0.1 ml enzyme extract. H _2_O _2_ was used to start the reaction, and a change in absorbance was detected at 470 nm.

**Determination of lipid peroxidation**

The lipid peroxidation was measured in the seedling stage by the amount of malonyl dialdehyde (MAD), as a product of peroxidation of unsaturated fatty acid (Linolenic acid, (18:3)). MAD concentration was estimated by the method of ( Heath & Packer, 1968). A sample of 0.5 g fresh leaves was extracted in 10 ml 5% (w/v) trichloroacetic acid. The homogenate was then centrifuged at 4000 rpm for 10 min. The supernatant (2 ml) was mixed with 2ml of 0.67% (w/v) thiobarbituric acid, incubated at 100°C in a water bath for 20 min then cooled immediately. Absorbance was read at 532 nm and 600 nm. MAD concentration (μM/g FW) was calculated using the extinction coefficient 155Mm ^-1^ cm ^-1^.

**Membrane leakage**

Fresh leaves were taken and quickly cut into sections measuring about 0.5 cm in length for the assessment of electrolyte leakage in the seedling and pre-flowering stages. After being submerged for one hour in 20 ml of distilled water, one-half g of these leaf fragments was used to test the electrical conductivity in the leaking solution.

**Mineral analysis of plant material**

We used half a gram of oven-dried plant samples. The sample was digested using a semi-a micro Kjeldahl apparatus and 2 mL of concentrated perchloric acid and 4 mL of concentrated sulfuric acid. Digestion continued until a clear solution without charging was obtained, after which it was filtered using Whatman filter paper NO. 44 and completed up to a constant volume. Phosphorus was determined using the Molybdenum blue method and measuring optical density at 700 nm, nitrogen using the Indo-phenol blue method according to ( Tetlow & Wilson, 1964) and measuring at 625 nm, and (K+) and (Na+) using the flame photometer ( Allen *et al.*, 1974). Protein nitrogen was calculated by the following equation:

Protein nitrogen (%)=N (%)×6.25.

**Determination of biogenic amines by thin-layer chromatography (TLC)**

Biogenic amines were extracted and determined in all tested samples according to Maijala and Eerola (1993).

**The yield stage**

Each barley cultivar was cultivated until the end of the growing season (starting from December 1 ^st^ 2010 until the end of April 2011). The yield criteria were measured, including the weight of grains per plant, the weight of 100 grains, and the number of grains per plant.

### Statistical analysis

All experimental determinations were replicated. The obtained data represented the mean values. Data obtained were analyzed statistically using excel to determine the degree of significance between treatments for each cultivar. The one-way ANOVA method was applied for all data and the least significant difference (LSD) at 0.05 and 0.01 was used to compare means ( Steel & Torrie, 1980).

## Results

### Seedling stage

**Figure 1.**
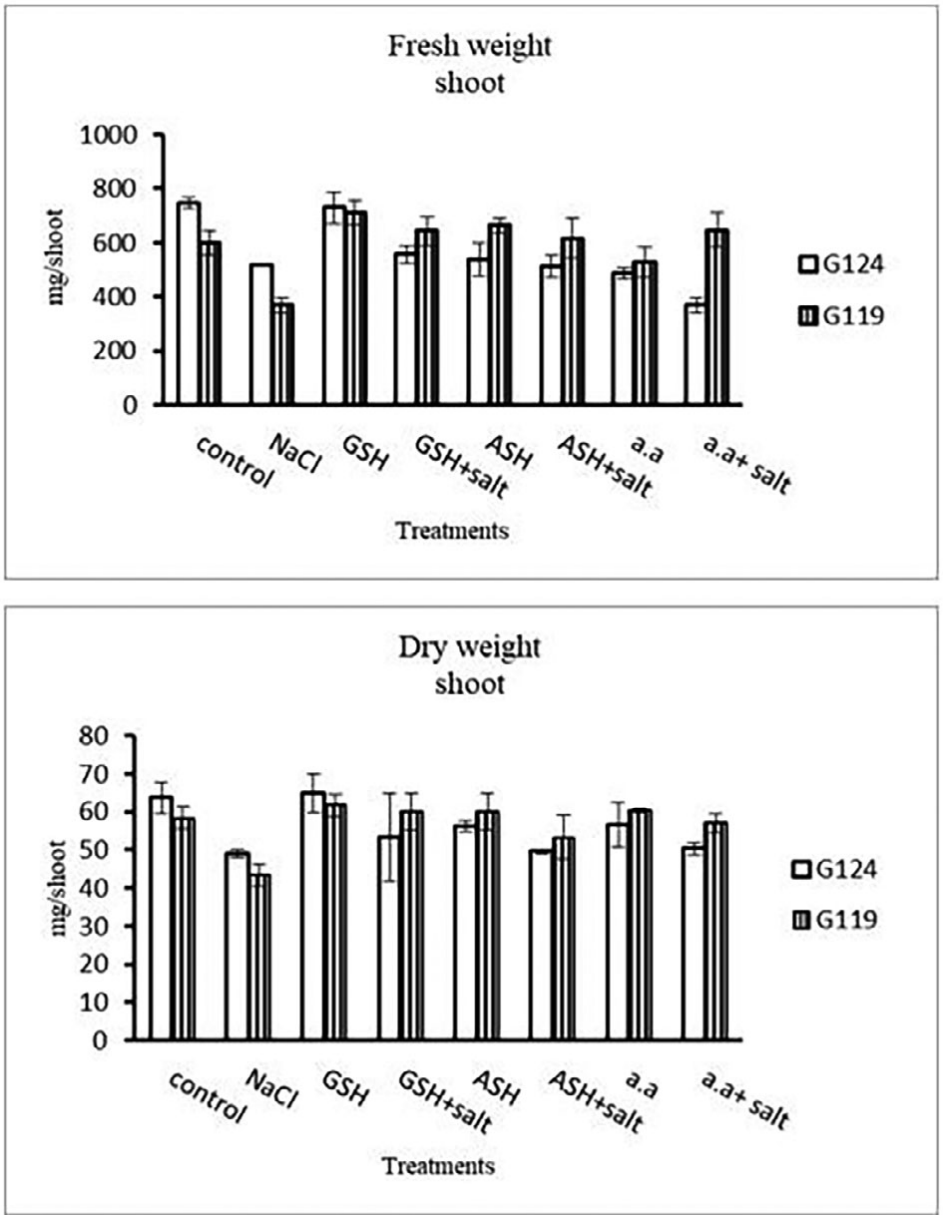
Effect of 100 mM NaCl with or without glutathione, ascorbic acid, or amino acid mixture on the shoot fresh and dry weights (mg/shoot) of two barley cultivars at the seedling stage.

**Figure 2.**
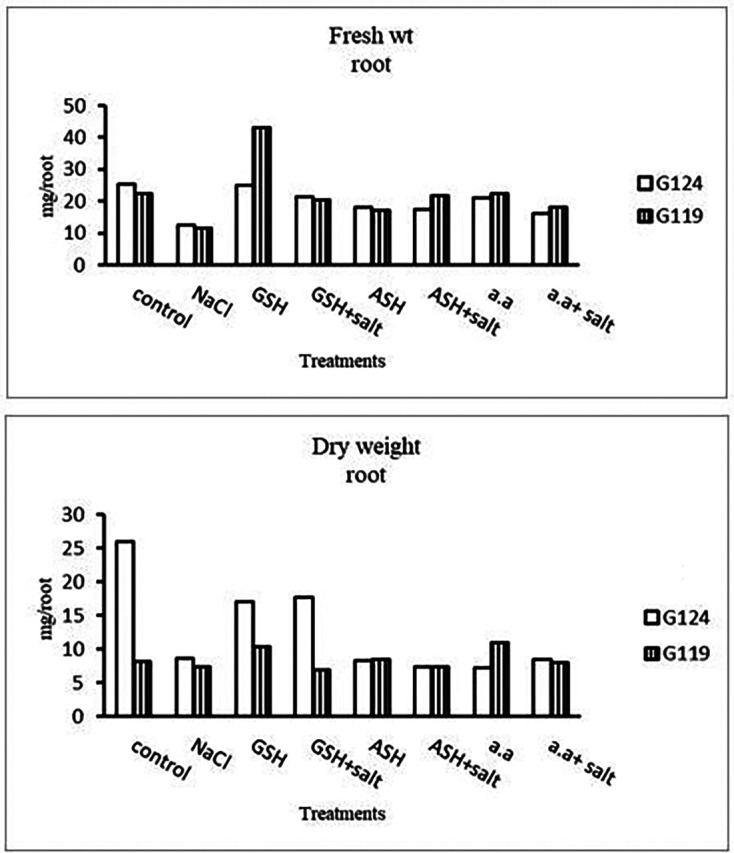
Effect of 100 mM NaCl with or without glutathione, ascorbic acid, or amino acid mixture on the root fresh and dry weights (mg/root) of two barley cultivars at the seedling stage.

**Figure 3.**
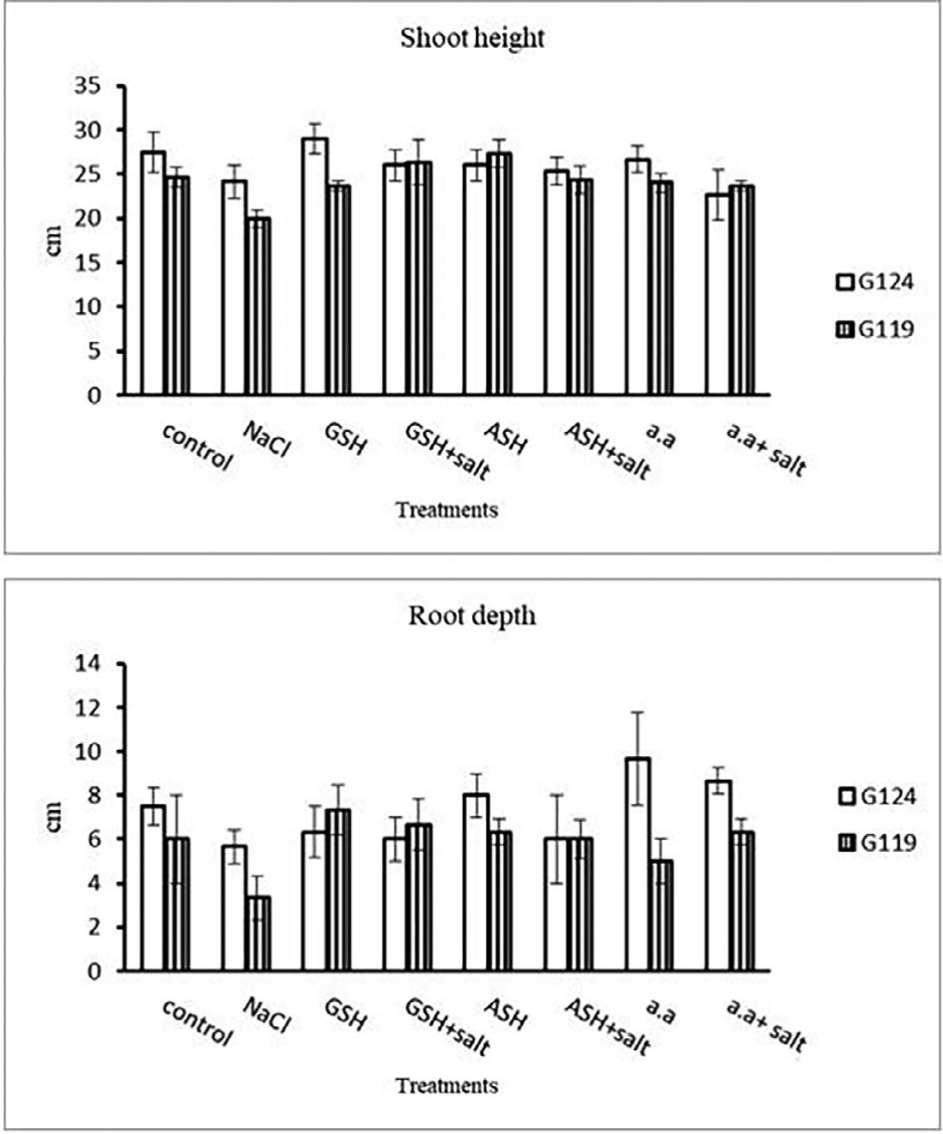
Effect of 100 mM NaCl with or without glutathione, ascorbic acid, or amino acid mixture on the shoot height (cm) and root depth (cm) of two barley cultivar at the seedling stage.

**Figure 4.**
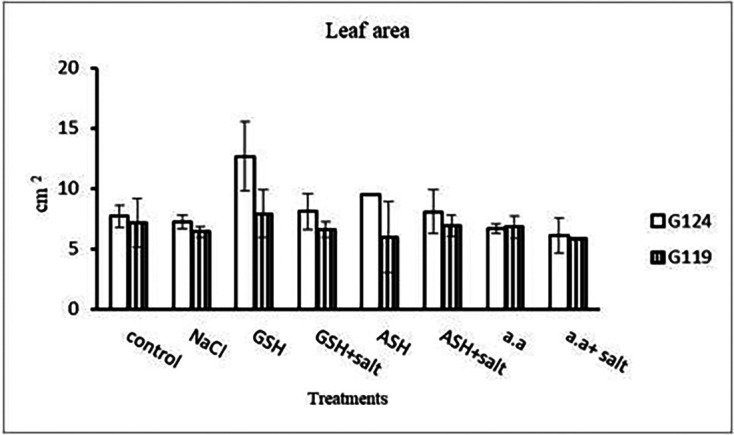
Effect of 100 mM NaCl with or without glutathione, ascorbic acid, or amino acid mixture on the leaf area (cm ^2^) of each barley cultivar at the seedling stage.

**Figure 5.**
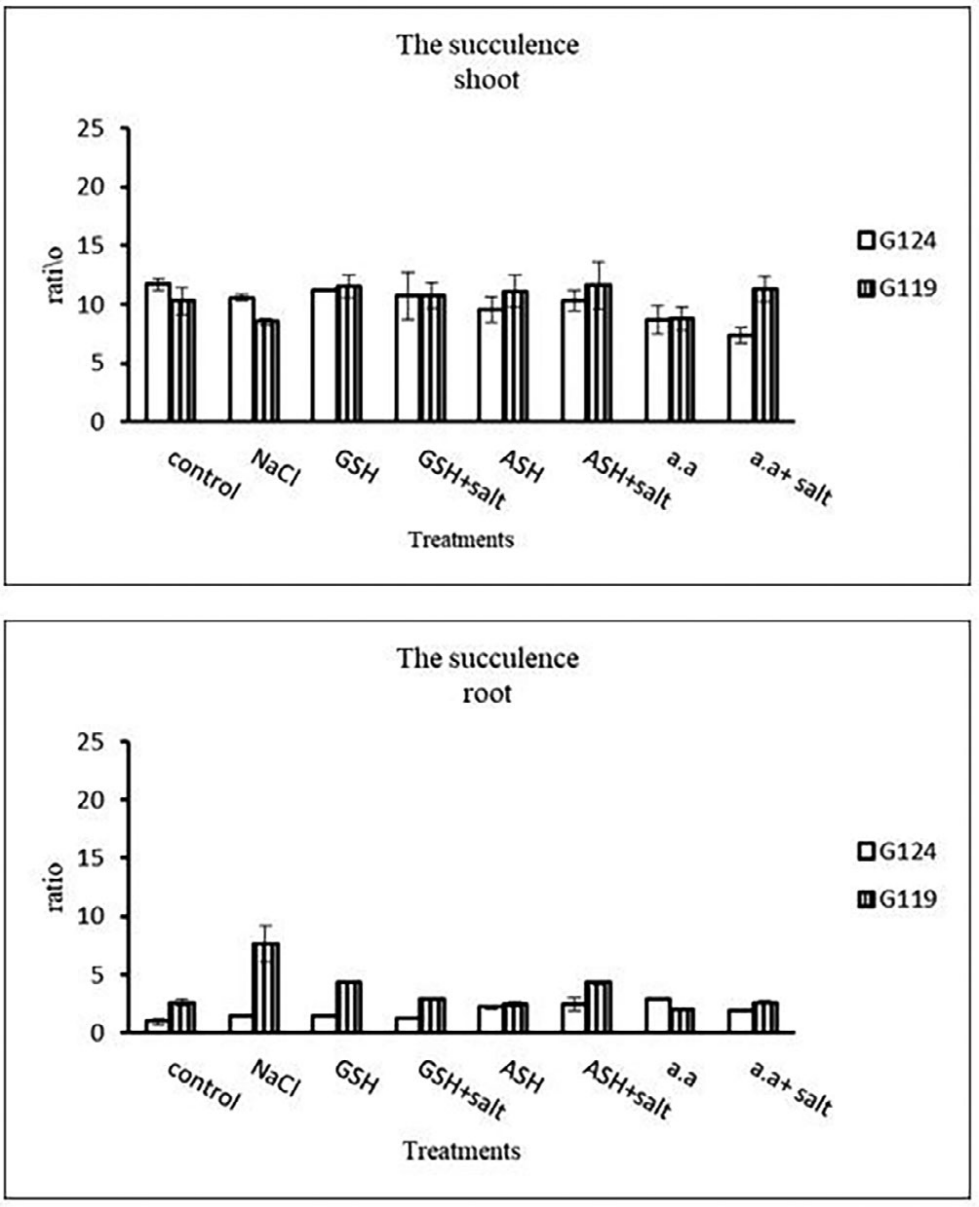
Effect of 100 mM NaCl with or without glutathione, ascorbic acid, or amino acid mixture on the shoot and root succulence of each barley cultivar at the seedling stage.

1- **Growth criteria**
  Grains of two barley cultivars, Giza 124, and Giza119 were germinated as described in the materials and methods section. Growth parameters (measured after 21 days), including fresh weight, dry weight, shoot height, root depth, and leaf area of barley seedlings were recorded as shown in Tables 1, 2, 3, and 4. The data showed that salinity stress caused a reduction in all studied growth parameters compared to the control. Also, it was observed that salinity resulted in a reduction in fresh and dry weights of both shoot and root of each cultivar compared to the control ( Table 1 and Figure 1). Increases in the fresh and dry weights of each cultivar's shoot and root following treatment with glutathione, ascorbic acid, or an amino acid combination offset the effects of salt. It is interesting to learn that, compared to other treatments, glutathione significantly increased the root dry weight of salt-affected Giza 124 ( Table 2 and Figure 2). It was observed that shoot height and root depth in each barley cultivar decreased under salinity compared to control ( Table 3 and Figure 3). However, shoot height increased in Giza 124 compared to Giza119 under the effect of glutathione, ascorbic acid or amino acid mixture. Moreover, root depth in Giza 124 reached its highest level under the effect of amino acid mixture compared to other treatments.
  Results revealed that in Giza 124, the leaf area expanded significantly under the influence of glutathione but to a lesser extent under the influence of ascorbic acid as compared to control and other treatments ( Table 4 and Figure 4), while glutathione showed a slight effect on Giza 119. The succulence ratio was decreased in the shoot and root of both cultivars with salinity but it increased in the root of Giza 119 under salinity and no variation was observed between other treatments ( Table 5 and Figure 5).

**Table 1.** Effect of 100 mM NaCl with or without glutathione, ascorbic acid or amino acid mixture on the fresh weight (mg/shoot) and dry weight (mg/shoot) of shoot of two barley cultivars at the seedling stage.

Treatments	Fresh weight		Dry weight	
	G124	G119	G124	G119
Control	746.3±18.6	599.6±47.4	63.7±04.2	58.3±2.8
NaCl	519.6±02.1	370.0±27.8	49.0±01.0	43.3±2.8
Glutathione	730.0±58.0	710.0±44.4	65.0±05.0	61.7±2.8
Glutathione plus salt	556.6±32.0	643.3±53.9	53.3±11.5	60.0±5.0
Ascorbic acid	540.0±61.0	663.3±27.5	56.3±1.53	60.0±5.0
Ascorbic acid plus salt	511.6±41.0	615.0±73.7	49.7±0.60	53.3±5.7
Amino acid mixtures	488.3±20.2	528.3±55.1	56.7±6.00	60.3±0.5
Amino acids plus salt	370.0±28.0	646.6±62.1	50.3±22.0	57.0±2.6
F	49.43	12.80	4.25	7.48
P	**	**	**	**
LSD	53.20	88.64	8.97	6.58

** Highly significant at P≤0.01.

**Table 2.** Effect of 100 mM of NaCl with or without glutathione, ascorbic acid or amino acid mixture on the fresh weight (mg/root) and dry weight (mg/root) of root of two barley cultivars at the seedling stage.

Treatments	Fresh weight		Dry weight	
	G124	G119	G124	G119
Control	25.3±0.001	22.3±0.002	26.0±0.001	8.2±0.001
NaCl	12.3±0.001	11.3±0.001	9.0±0.001	7.4±0.001
Glutathione	25.0±0.001	43.0±0.001	17.0±0.001	10.3±0.001
Glutathione plus salt	21.3±0.001	20.3±0.001	17.7±0.001	7.0±0.001
Ascorbic acid	18.0±0.001	17.0±0.001	8.3±0.001	8.5±0.001
Ascorbic acid plus salt	17.3±0.001	22.0±0.001	7.5±0.001	7.3±0.001
Amino acid mixtures	21.0±0.001	22.3±0.001	7.3±0.001	11.0±0.001
Amino acids plus salt	16.0±0.001	18.0±0.001	8.6±0.001	8.0±0.001
F	8.25	288.2	193.01	21311.5
P	**	**	**	**
LSD	4.67	2.09	1.48	6.11

** Highly significant at P≤0.01.

**Table 3.** Effect of 100 mM NaCl with or without glutathione, ascorbic acid or amino acid mixture on the shoot height (cm) and root depth (cm) of two barley cultivars at the seedling stage.

Treatments	Shoot height		Root depth	
	G124	G119	G124	G119
Control	27.5 ±2.3	24.7 ± 1.1	7.5 ±0.9	6.0 ±2.0
NaCl	24.2 ±1.9	20.0 ± 1.0	5.7 ±0.8	3.3 ±1.0
Glutathione	29.0 ±1.7	23.7 ± 0.5	6.3 ±1.2	7.3 ±1.2
Glutathione plus salt	26.0 ±1.7	26.3 ± 2.5	6.0 ±1.0	6.7 ±1.2
Ascorbic acid	26.0 ±1.7	27.3 ± 1.5	8.0 ±1.0	6.3 ±0.6
Ascorbic acid plus salt	25.3±1.5	24.3 ± 1.5	6.0 ±2.0	6.0 ±0.9
Amino acid mixtures	26.7 ±1.5	24.0 ± 1.0	9.7 ±2.1	5.0 ±1.0
Amino acids plus salt	22.7 ±2.0	23.7± 0.5	8.7 ±0.6	6.3 ±0.6
F	2.96	7.50	3.72	2.161
P	*	**	*	ns
LSD	3.38	2.37	2.37	1.94

* Significant at P≤0.05.

** Highly significant at P≤0.01.

**Table 4.** Effect of 100 mM NaCl with or without glutathione, ascorbic acid, or amino acid mixture on the leaf area (cm ^2^) of two barley cultivars at the seedling stage.

Treatments	Leaf area	
	G124	G119
Control	7.7±0.9	7.1 ±1.99
NaCl	7.2±0.6	6.4±0.45
Glutathione	12.7 ±7.9	7.9±1.96
Glutathione plus salt	8.1±1.5	6.6±0.62
Ascorbic acid	9.5±0.1	5.9±2.96
Ascorbic acid plus salt	8.1±1.9	6.9±0.90
Amino acid mixtures	6.7±0.4	6.8±0.93
Amino acids plus salt	6.1±1.5	5.8±0.14
F	5.82	0.56
P	**	ns
LSD	2.54	2.66

** Highly significant at P≤0.01.

**Table 5.** Effect of 100 mM NaCl with or without glutathione, ascorbic acid, or amino acid mixture on the shoot and root succulence of two barley cultivars at the seedling stage.

Treatments	Succulence			
	Shoot		Root	
	G124	G119	G124	G119
Control	11.7±0.5	10.3±1.1	0.9±0.3	2.6±0.3
NaCl	10.6±0.3	8.5±0.2	1.4±0.1	7.6±1.5
Glutathione	11.2±0.1	11.5±1.0	1.5±0.1	4.3±0.1
Glutathione plus salt	10.7±1.9	10.8±1.1	1.2±0.1	2.9±0.1
Ascorbic acid	9.6 ±1.2	11.1±1.4	2.2±0.2	2.4±0.3
Ascorbic acid plus salt	10.3±0.9	11.6±2.0	2.4±0.6	4.3±0.2
Amino acid mixtures	8.7±1.2	8.8±0.9	2.9±0.1	22.3±0.6
Amino acids plus salt	7.4±0.6	11.3±1.1	1.9±0.1	2.6±0.1
F	5.94	2.99	20.30	31.38
P	**	*	**	**
LSD	1.74	2.10	0.43	0.98

ns=not significant.

* Significant at P≤0.05.

** Highly significant at P≤0.01.

Statistical analysis showed that the effects of salinity, glutathione, ascorbic acid or amino acid mixture with or without salt were highly significant (P≤0.01) on each studied growth parameter compared to the control of each cultivar.

2- **Photosynthesis**

**Figure 6.**
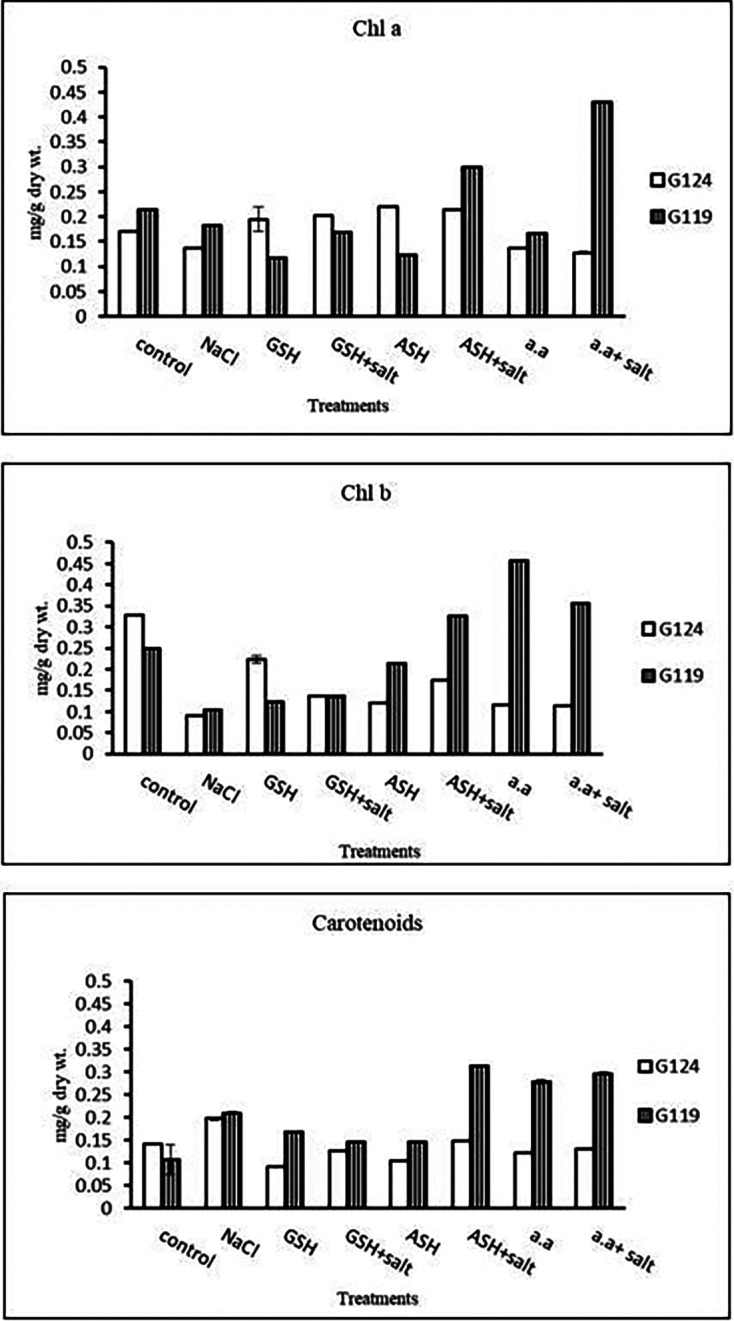
Effect of 100 mM NaCl with or without glutathione, ascorbic acid, or amino acid mixture on chlorophyll a, chlorophyll b and carotenoids of two barley cultivars at the seedling stage.

**Figure 7.**
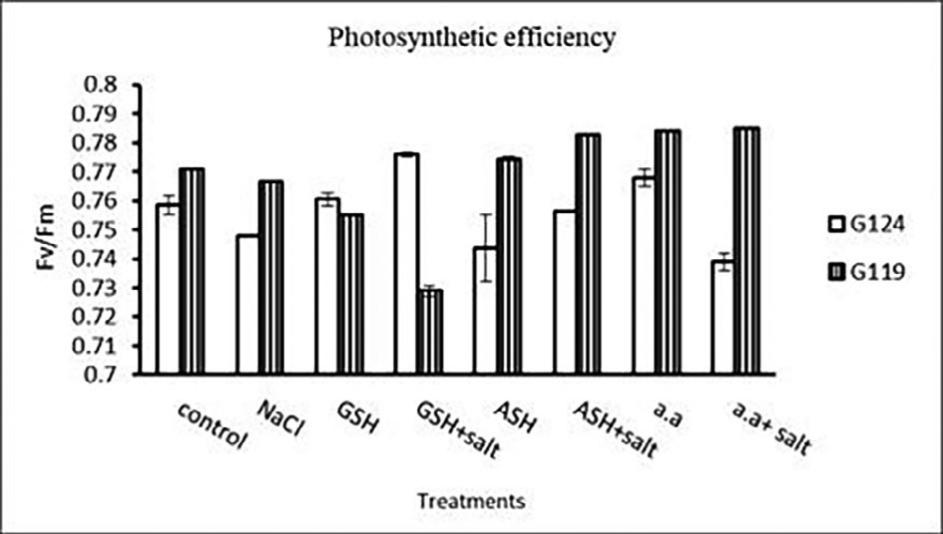
Effect of 100 mM NaCl with or without glutathione, ascorbic acid, or amino acid mixture on photosynthetic efficiency of two barley cultivars at the seedling stage.

a) **Chlorophyll contents:** Regarding Giza 119, a remarkable rise of all determined pigments can be observed with the addition of either ascorbic acid or amino acid mixture to salt-stressed seedlings. However, the effect was more pronounced in chl b ( Table 6 and Figure 6). Again, Giza 119 showed higher values of chl a, chl b, and carotenoids with the addition of ascorbic acid or amino acid mixture to salt-stressed seedlings and the difference were greater between the two cultivars. However, no remarkable effect of glutathione was observed with salt on most pigments.
b) **Photosynthetic efficiency:** The addition of either glutathione, ascorbic acid or amino acid mixture with salt showed greater photosynthetic efficiency for each cultivar but the effect was greater with Giza 119 compared to Giza 124, which can be correlated with the pigment content ( Table 7 and Figure 7).

**Table 6.** Effect of 100 mM NaCl with or without glutathione, ascorbic acid, or amino acid mixture on photosynthetic pigments of two barley cultivars at the seedling stage.

Treatments	Chlorophyll a		Chlorophyll b		Carotenoids	
	G124	G119	G 124	G119	G 124	G119
Control	0.17±0.0	0.21±0.0	0.33±0.0	0.25±0.0	0.14±0.0	0.11±0.0
NaCl	0.13±0.0	0.11±0.0	0.09±0.0	0.12±0.0	0.20±0.0	0.21±0.0
Glutathione	0.19±0.0	0.13±0.0	0.22±0.0	0.13±0.0	0.09±0.0	0.17±0.0
Glutathione plus salt	0.20±0.0	0.17±0.0	0.14±0.0	0.14±0.0	0.13±0.0	0.15±0.0
Ascorbic acid	0.22±0.0	0.18±0.0	0.12±0.0	0.19±0.0	0.10±0.0	0.14±0.0
Ascorbic acid plus salt	0.21±0.0	0.19±0.0	0.18±0.0	0.27±0.0	0.15±0.0	0.32±0.0
Amino acid mixtures	0.14±0.0	0.17±0.0	0.12±0.0	0.33±0.0	0.12±0.0	0.28±0.0
Amino acids plus salt	0.13±0.0	0.18±0.0	0.11±0.0	0.30±0.0	0.13±0.0	0.30±0.0
F	53.91	3284.81	1212.78	2082.19	2459.19	135.57
P	**	**	**	**	**	**
LSD	0.02	1.69	6.76	5.43	1.93	0.020

** Highly significant at P≤0.01.

**Table 7.** Effect of 100 mM NaCl with or without glutathione, ascorbic acid, or amino acid mixture on photosynthetic efficiency of two barley cultivars at the seedling stage.

Treatments	Photosynthetic efficiency	
	G124	G119
Control	0.75±0.003	0.77±0.001
NaCl	0.74±0.002	0.76±0.001
Glutathione	0.76±0.002	0.75±0.001
Glutathione plus salt	0.77±0.001	0.72±0.002
Ascorbic acid	0.74±0.011	0.77±0.005
Ascorbic acid plus salt	0.75±0.001	0.78±0.001
Amino acid mixtures	0.76±0.003	0.78±0.001
Amino acids plus salt	0.73±0.003	0.78±0.002
F	9.06	1844.2
P	**	**
LSD	0.011	1.32

** Highly significant at P≤0.01.

Statistical analysis showed that the effects of salinity, glutathione, ascorbic acid, or amino acid mixture with or without salt were highly significant (P≤0.01) on chlorophyll contents and photosynthetic efficiency of each cultivar.

3- **Metabolites**

**Figure 8.**
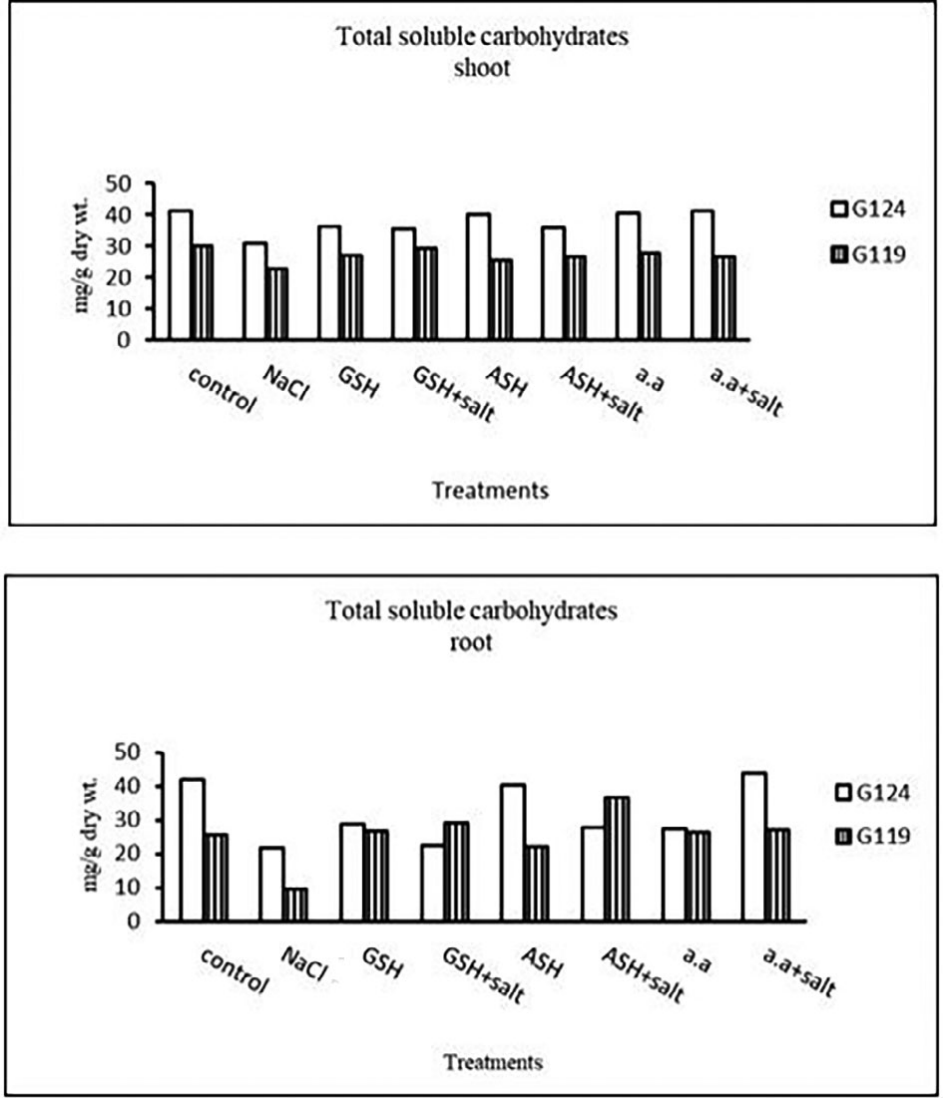
Effect of 100 mM NaCl with or without glutathione, ascorbic acid, or amino acid mixture on T.S.C (mg/g dry wt.) in two barley cultivars.

**Figure 9.**
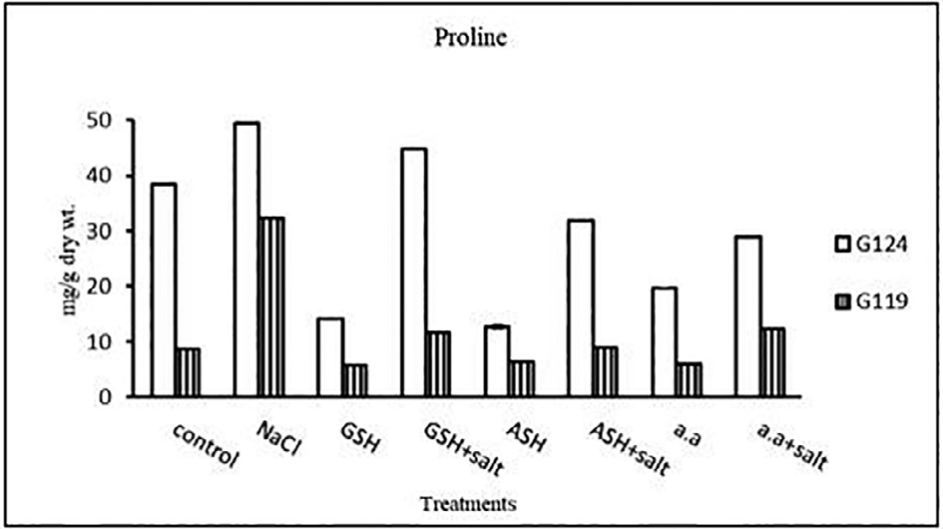
Effect of 100 mM NaCl with or without glutathione, ascorbic acid, or amino acid mixture on proline content (mg/g dry wt.) in two barley cultivars.

a) **Total soluble carbohydrates:** In each cultivar, there was a decrease in total soluble carbohydrates in shoot and root under salinity compared to the control ( Table 8 and Figure 8). With the addition of glutathione, ascorbic acid, or amino acid mixture to salt total soluble carbohydrates increased.
b) **Proline content:** In both cultivars Salinity has resulted in an increase in proline content compared with the control and the effect was more pronounced in Giza 124 than Giza 119 ( Table 9 and Figure 9). However, proline content decreased with glutathione, ascorbic acid, or amino acid mixture compared to control. Proline content decreased also with the addition of each of these compounds to salt compared with salt alone.

**Table 8.** Effect of 100 mM NaCl with or without glutathione, ascorbic acid, or amino acid mixture on total soluble carbohydrates (mg/g dry wt.) of shoot and root of two barley cultivars at the seedling stage.

Treatments	Shoot		Root	
	G124	G119	G124	G119
Control	41.3±0.0	30.0±0.1	41.9±0.0	25.6±0.2
NaCl	30.9 ±0.1	22.7±0.2	21.8±0.0	9.6±0.1
Glutathione	36.3±0.0	26.9±0.1	28.9±0.0	26.7±0.1
Glutathione plus salt	35.4±0.0	29.2±0.0	22.5±0.0	29.2±0.0
Ascorbic acid	40.0±0.1	25.5±0.1	40.1±0.0	22.1±0.1
Ascorbic acid plus salt	35.7±0.2	26.6±0.1	27.8±0.0	36.6±0.0
Amino acid mixtures	40.4±0.0	27.9±0.0	27.5±0.0	26.2±0.1
Amino acids plus salt	41.3±0.0	26.6±0.0	43.9±0.0	27.2±0.0
F	2347.45	1316.51	19213.68	21155.64
P	**	**	**	**
LSD	0.22	0.18	0.19	0.15

** Highly significant at P≤0.01.

**Table 9.** Effect of 100 mM NaCl with or without glutathione, ascorbic acid, or amino acid mixture on proline content (mg/g dry wt.) of two barley cultivars at the seedling stage.

Treatments	Proline	
	G 124	G119
Control	38.4±0.1	8.7±0.1
NaCl	49.3±0.0	32.2±0.1
Glutathione	14.2±0.1	5.7±0.1
Glutathione plus salt	44.8±0.0	11.8±0.1
Ascorbic acid	12.7±0.4	6.3±0.1
Ascorbic acid plus salt	31.9±0.1	8.9±0.1
Amino acid mixtures	19.8±0.0	5.9±0.1
Amino acids plus salt	28.9±0.1	12.4±0.1
F	22416.1	39589.9
P	**	**
LSD	0.27	0.13

** Highly significant at P≤0.01.

Statistical analysis showed that the effects of salinity, glutathione, ascorbic acid, or amino acid mixture with or without salt were highly significant (P≤0.01) the on total soluble carbohydrates and proline content of each cultivar.

**Figure 10.**
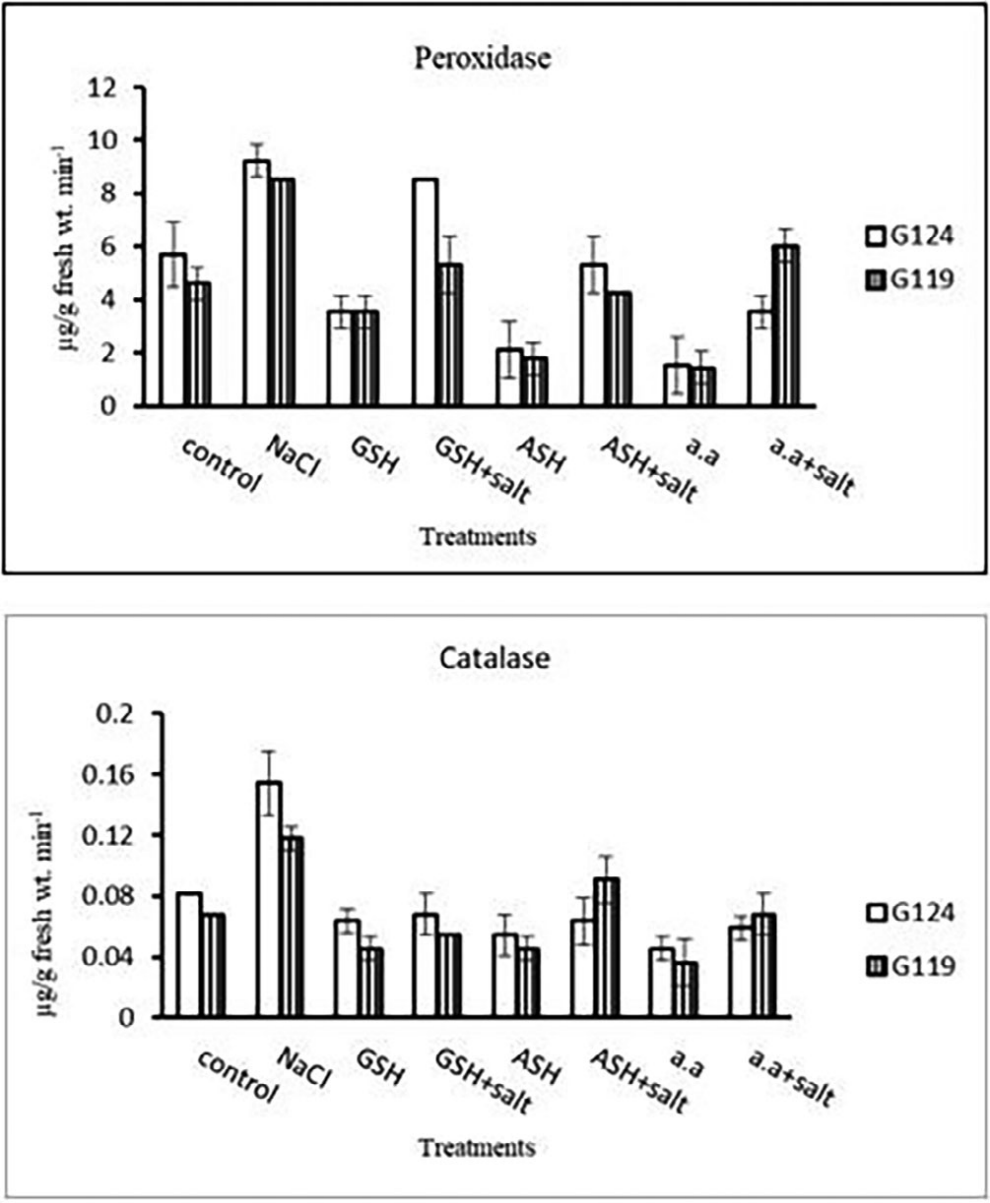
Effect of 100 mM NaCl with or without glutathione, ascorbic acid, or amino acid mixture on peroxidase and catalase (μg/g fresh wt. min ^-1^) of two barley cultivars at the seedling stage.

**Figure 11.**
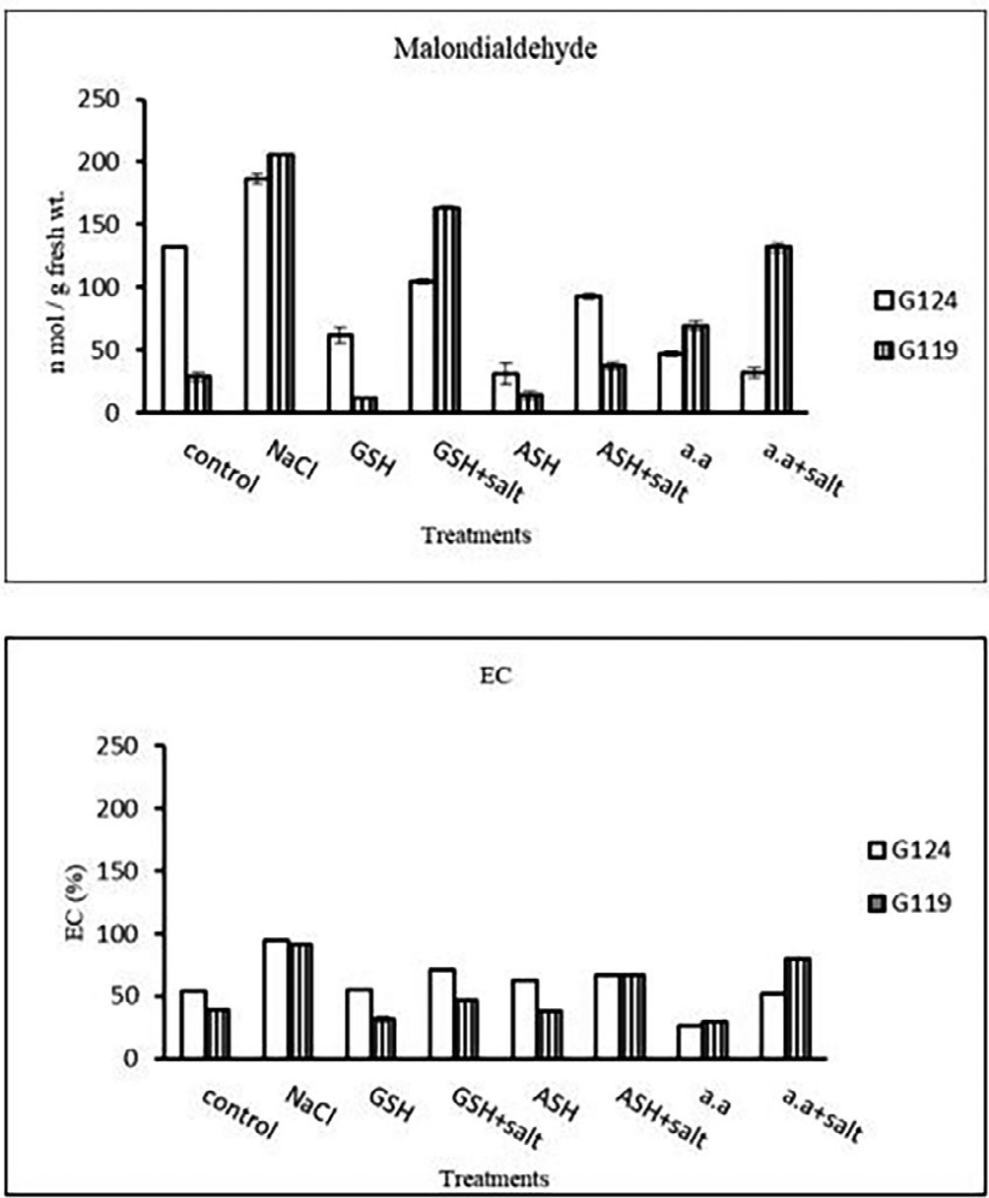
Effect of 100 mM NaCl with or without glutathione, ascorbic acid, or amino acid mixture on malondialdehyde (MDA) content (n mol/g fresh wt.) and membrane leakage (EC%) of two barley cultivars at the seedling stage.

4- **Antioxidant enzymes:** Results indicated that in each cultivar the activity of peroxidase and catalase increased under the effect of salinity compared to the control. The effect of salinity on peroxidase and catalase decreased with the addition of glutathione, ascorbic acid, or amino acid mixture ( Table 10 and Figure 10).
5- **Malondialdehyde content:** In each cultivar, salinity showed an increase in malondialdehyde (MAD) content compared to the control. However, lipid peroxidation has decreased with glutathione, ascorbic acid, or amino acid mixture compared to the control. Giza 119 showed more MDA content compared to Giza 124 under salinity ( Table 11 and Figure 11).
6- **Membrane leakage:** The results of both cultivars were more or less similar. However, salinity stress increased the membrane leakage of Giza 124 and Giza 119, while decreased by the addition of glutathione, ascorbic acid or amino acid mixture ( Table 11 and Figure 11).

**Table 10.** Effect of 100 mM NaCl with or without glutathione, ascorbic acid, or amino acid mixture on peroxidase and catalase (μg/g fresh wt. min ^-1^) of two barley cultivars at the seedling stage.

Treatments	Peroxidase		Catalase	
	G124	G119	G124	G119
Control	5.7±1.2	4.6±0.6	0.08±0.0	0.07±0.0
NaCl	9.2±0.6	8.5±0.0	0.15±0.0	0.12±0.0
Glutathione	3.5±0.6	3.6±0.6	0.06±0.0	0.05±0.0
Glutathione plus salt	8.5±0.1	5.3±1.1	0.07±0.0	0.05±0.0
Ascorbic acid	2.1±1.1	1.8±0.6	0.05±0.0	0.05±0.0
Ascorbic acid plus salt	5.3±1.1	4.2±0.0	0.06±0.0	0.09±0.0
Amino acid mixtures	1.5±1.0	1.4±0.6	0.05±0.0	0.04±0.0
Amino acids plus salt	3.5±0.6	6.0±0.6	0.06±0.0	0.07±0.0
F	31.59	42.11	22.68	20.50
P	**	**	**	**
LSD	1.49	1.06	0.021	0.017

** Highly significant at P≤0.01.

**Table 11.** Effect of 100 mM NaCl with or without glutathione, ascorbic acid, or amino acid mixture on malondialdehyde content (n mol/g fresh wt.) and membrane leakage (EC%) of two barley cultivars at the seedling stage.

Treatments	Malondialdehyde content		Membrane leakage	
	G124	G119	G124	G119
Control	132.5±1.2	28.7±3.7	54.2±0.2	38.8±0.4
NaCl	186.3±4.0	205.8±0.0	94.1±0.2	91.1±0.1
Glutathione	62.0±6.4	11.6±1.1	54.9±0.8	31.6±0.9
Glutathione plus salt	104.6±2.0	163.5±1.7	71.1±0.7	47.0±0.0
Ascorbic acid	31.0±8.9	13.9±3.4	62.3±0.6	38.3±0.5
Ascorbic acid plus salt	93.0±2.3	37.2±3.0	67.2±0.3	67.3±0.3
Amino acid mixtures	46.9±2.1	69.8±3.4	25.9±0.1	29.3±0.0
Amino acids plus salt	32.2±4.1	131.7±3.7	51.6±0.4	79.3±0.5
F	437.83	2959.37	4707.67	2977.99
P	**	**	**	**
LSD	7.78	5.03	0.84	1.28

** Highly significant at P≤0.01.

Statistical analysis showed that in each cultivar the effects of salinity, glutathione, ascorbic acid or amino acid mixture with or without salt were highly significant (P≤0.01) on the activity of each enzyme, MDA content and the membrane leakage of each cultivar.

7- **Mineral analysis**

**Figure 12.**
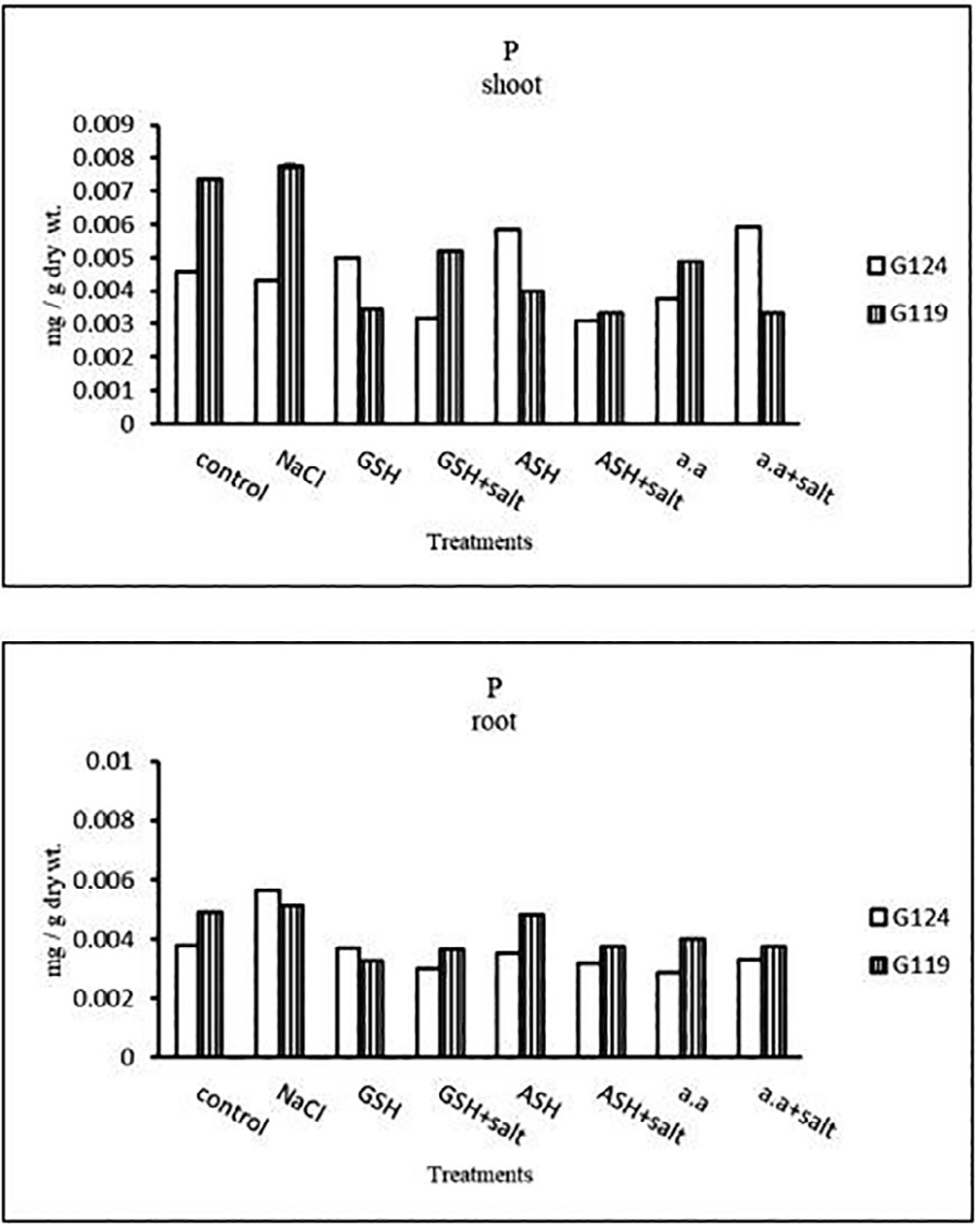
Effect of 100 mM NaCl with or without glutathione, ascorbic acid, or amino acid mixture on phosphorus content (mg/g dry wt.) of shoot and root of two barley cultivars at the seedling stage.

**Figure 13.**
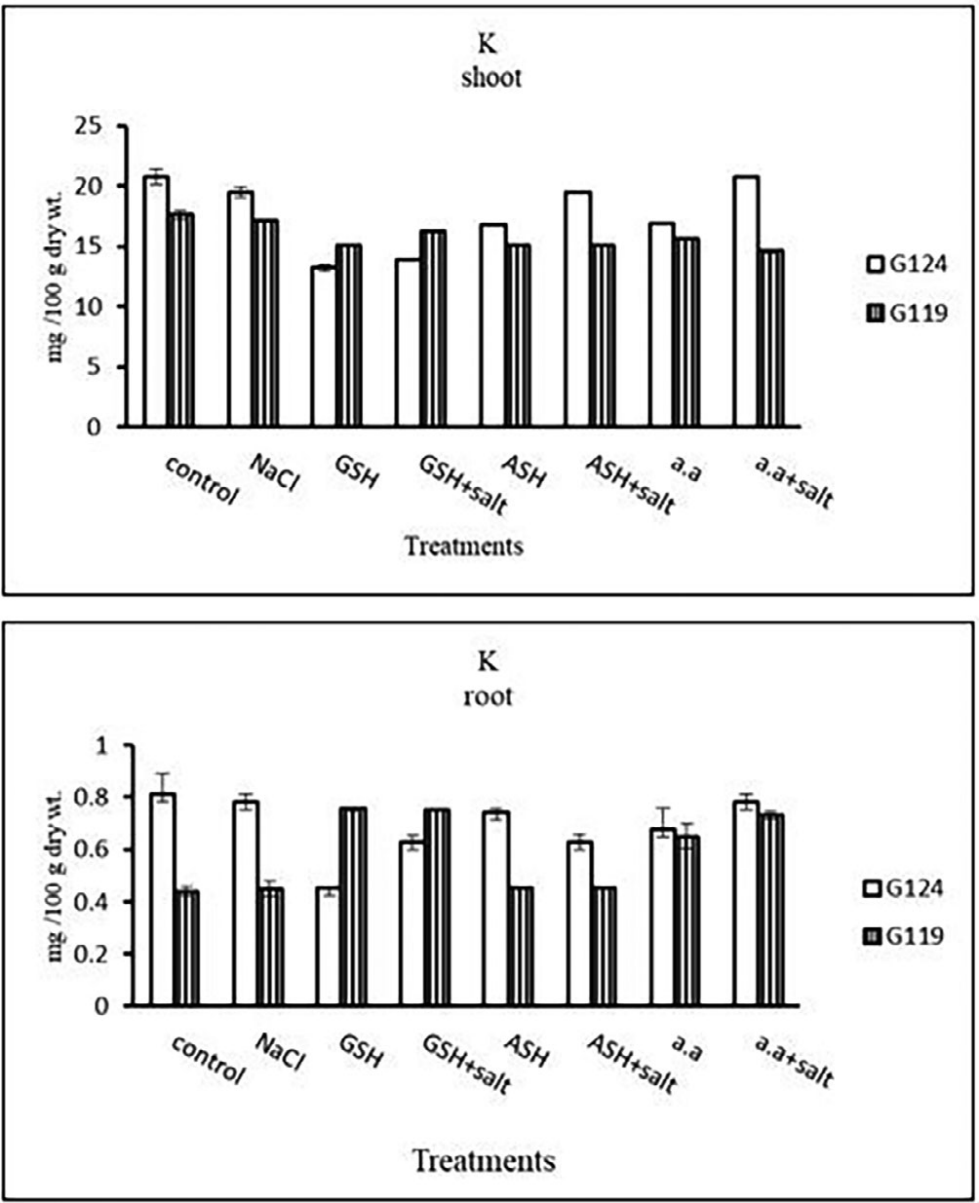
Effect of 100 mM NaCl with or without glutathione, ascorbic acid, or amino acid mixture on potassium content (mg/100 g dry wt.) of shoot and root of two barley cultivars at the seedling stage.

**Figure 14.**
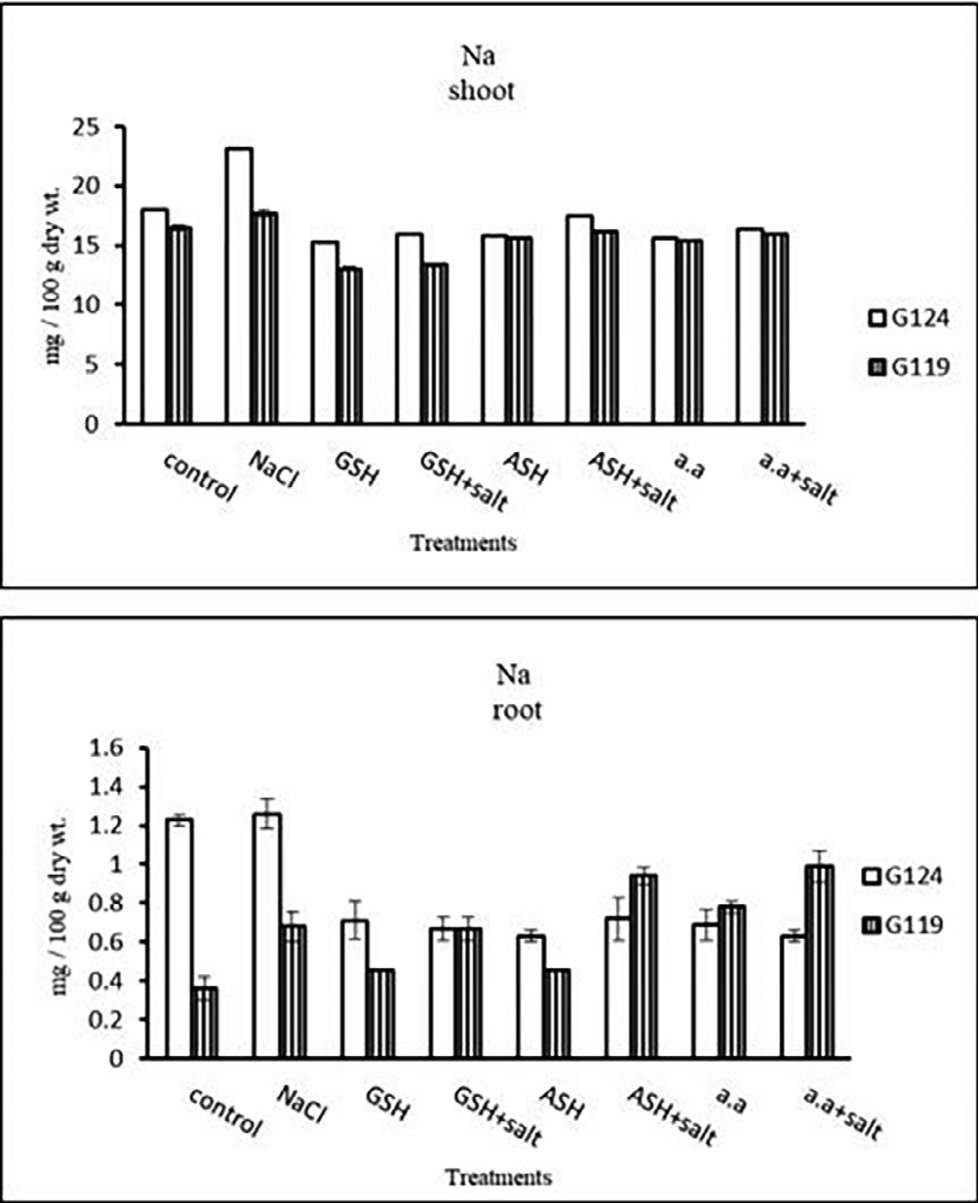
Effect of 100 mM NaCl with or without glutathione, ascorbic acid, or amino acid mixture on sodium content (mg/100 g dry wt.) of shoot and root of two barley cultivars at the seedling stage.

**Figure 15.**
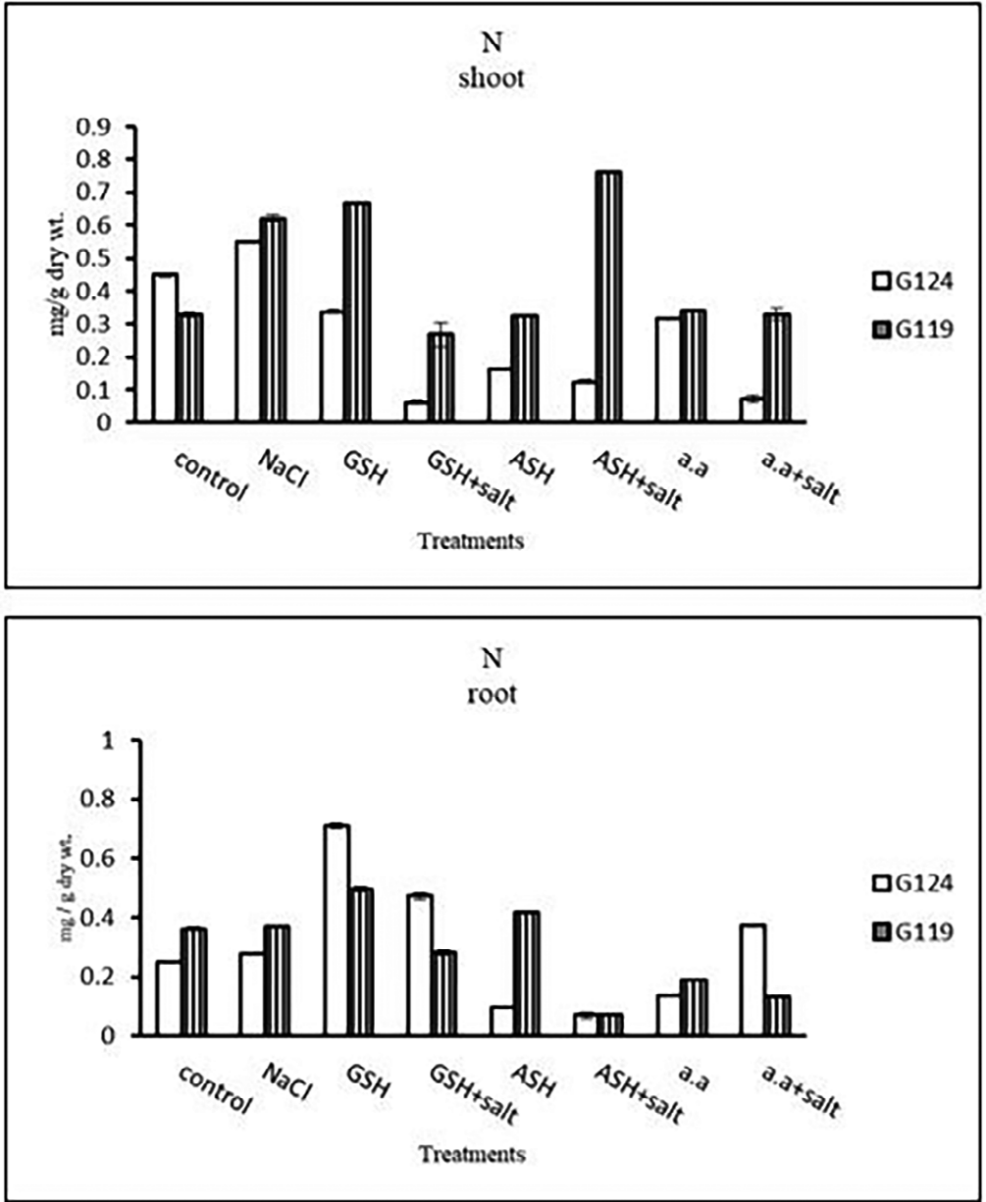
Effect of 100 mM NaCl with or without glutathione, ascorbic acid, or amino acid mixture on nitrogen content (mg/g dry wt.) of shoot and root of two barley cultivars at the seedling stage.

**Figure 16.**
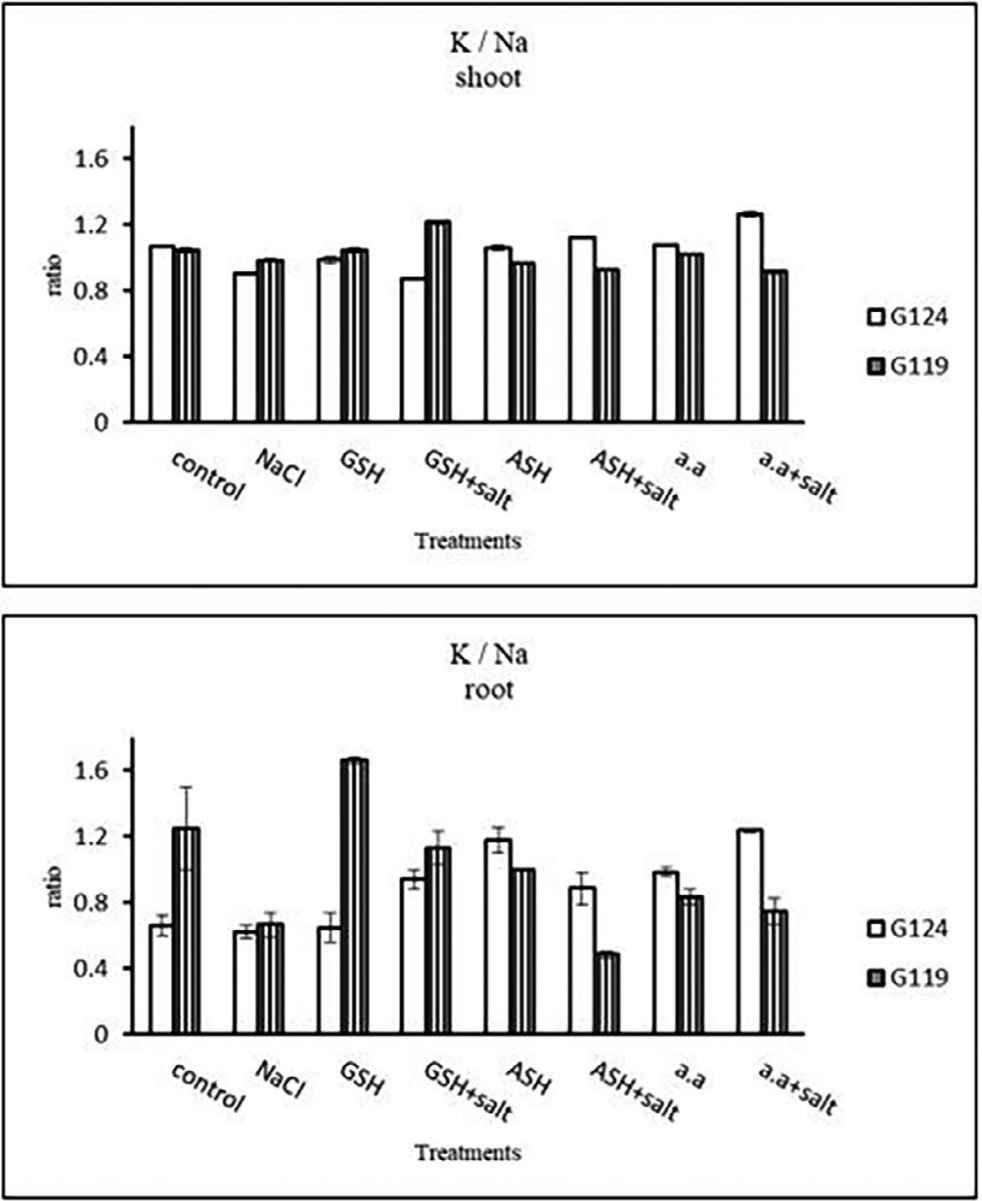
Effect of 100 mM NaCl with or without glutathione, ascorbic acid, or amino acid mixture on K/Na ratio of shoot and root of two barley cultivars at the seedling stage.

**Figure 17.**
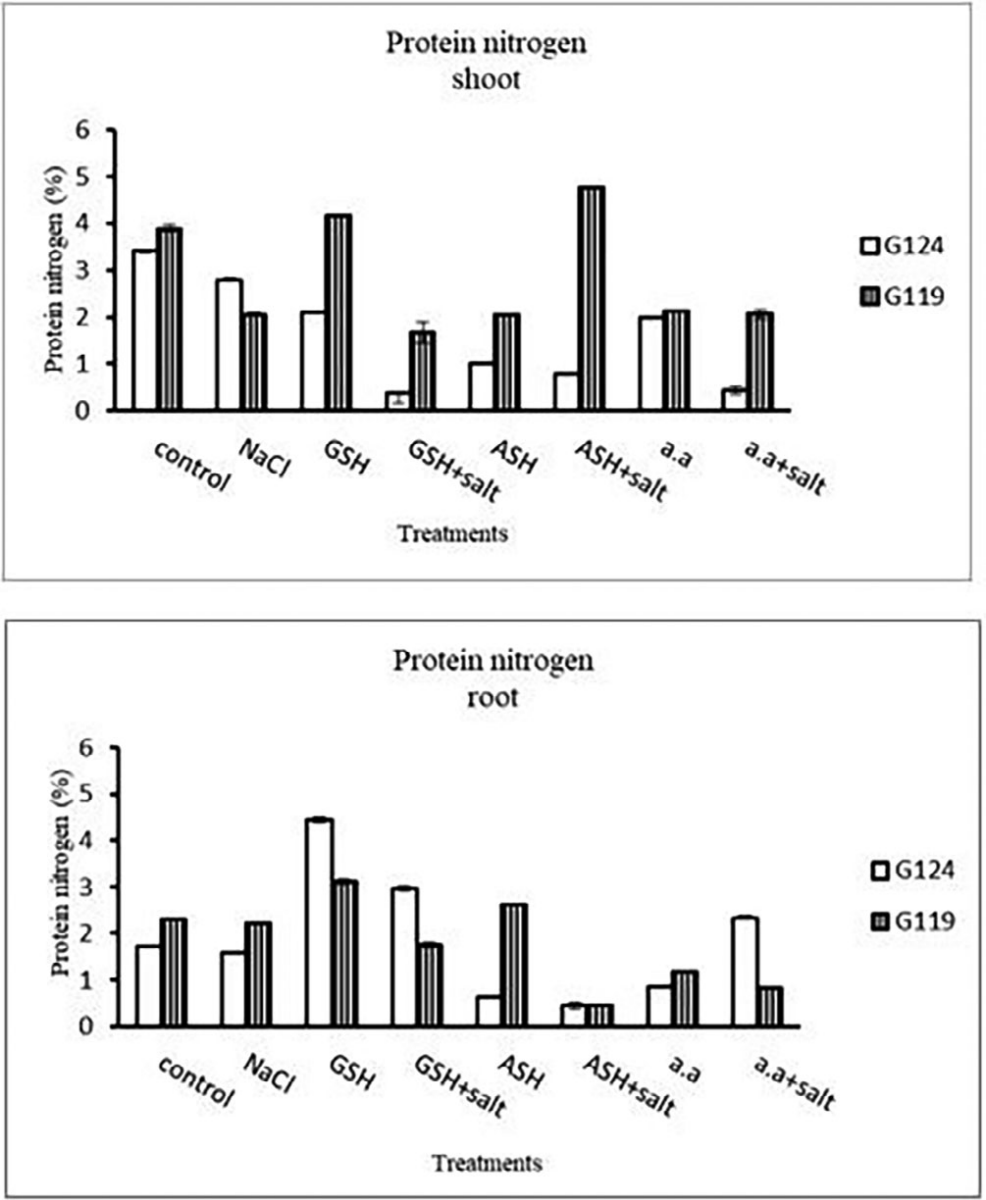
Effect of 100 mM NaCl with or without glutathione, ascorbic acid, or amino acid mixture on protein nitrogen (%) of shoot and root of two barley cultivars at the seedling stage.

a) **Phosphorus content:** Salinity showed a slight increase in phosphorus content in the shoot and root of both cultivars ( Table 12 and Figure 12). However, it increased in Giza 124 shoot under the effect of glutathione, ascorbic acid without salt or amino acid mixture plus salt. On the other hand, it decreased in Giza 124 root under the effect of glutathione, ascorbic acid, or amino acid mixture with or without salt compared to the control, while increased in Giza 119 root under the effect of ascorbic acid alone.
b) **Potassium content:** It can be noticed that potassium showed a decrease in the shoot and root of each cultivar, especially with salinity alone ( Table 13 and Figure 13). However, Giza 124 shoot has experienced a rise in potassium content in most treatments and showed lower root potassium with salinity alone and ascorbic acid with or without salt or amino acids with salt compared to Giza 119.
c) **Sodium content:** Results showed that sodium content increased under salinity in the shoot and root of both cultivars compared to the control ( Table 14 and Figure 14). However it increased with glutathione, ascorbic acid, or amino acids with the salt in the root of Giza 119. Potassium/sodium ratio decreased with salt, while it has been increased with other treatments compared to the control ( Table 16 and Figure 16).
d) **Nitrogen content:** Results showed that nitrogen content increased in shoots of both Giza 124 and 119 under the effect of salinity compared to the control ( Table 15 and Figure 15). However, it increased in the shoot and root of each cultivar under the effect of glutathione compared to other treatments.
e) **Protein nitrogen:** Shoot protein nitrogen showed a decrease with salt in both cultivars and appreciably with glutathione, ascorbic acid, or amino acid plus salt in Giza 124 compared to other treatments ( Table 17 and Figure 17). In both cultivars, root-protein nitrogen showed a remarkable increase with glutathione alone in Giza124 and Giza 119.

**Table 12.** Effect of 100 mM NaCl with or without glutathione, ascorbic acid, or amino acid mixture on phosphorus content (mg/g dry wt.) of shoot and root of two barley cultivars at the seedling stage.

Treatments	Phosphorus			
	Shoot		Root	
	G124	G119	G124	G119
Control	0.004±0.0	0.008±0.0	0.006±0.0	0.005±0.0
NaCl	0.005±0.0	0.007±0.0	0.004±0.0	0.004±0.0
Glutathione	0.005±0.0	0.003±0.0	0.004±0.0	0.003±0.0
Glutathione plus salt	0.003±0.0	0.005±0.0	0.003±0.0	0.004±0.0
Ascorbic acid	0.006±0.0	0.004±0.0	0.004±0.0	0.005±0.0
Ascorbic acid plus salt	0.003±0.0	0.003±0.0	0.003±0.0	0.004±0.0
Amino acid mixtures	0.004±0.0	0.005±0.0	0.003±0.0	0.004±0.0
Amino acids plus salt	0.006±0.0	0.003±0.0	0.003±0.0	0.004±0.0
F	4294.7	7480.7	1105.7	1725.6
P	**	**	**	**
LSD	4.99	6.12	7.90	4.99

** Highly significant at P≤0.01.

**Table 13.** Effect of 100 mM NaCl with or without glutathione, ascorbic acid, or amino acid mixture on potassium content (mg/100 g dry wt.) of shoot and root of two barley cultivars at the seedling stage.

Treatments	Potassium			
	Shoot		Root	
	G124	G119	G124	G119
Control	20.8±0.7	17.6±0.3	0.8±0.1	0.4±0.0
NaCl	19.5±0.5	17.1±0.0	0.7±0.0	0.4±0.0
Glutathione	13.2±0.3	15.1±0.2	0.4±0.0	0.7±0.0
Glutathione plus salt	13.8±0.0	16.8±0.1	0.6±0.0	0.7±0.0
Ascorbic acid	16.8±0.0	15.1±0.1	0.7±0.0	0.4±0.0
Ascorbic acid plus salt	19.5±0.0	15.1±0.2	0.6±0.0	0.4±0.0
Amino acid mixtures	16.9±0.1	15.7±0.1	0.7±0.1	0.6±0.1
Amino acids plus salt	20.8±0.1	14.6±0.2	0.7±0.0	0.7±0.0
F	21.9	177.0	22.2	147.9
P	**	**	**	**
LSD	0.07	0.03	0.25	0.12

** Highly significant at P≤0.01.

**Table 14.** Effect of 100 mM NaCl with or without glutathione, ascorbic acid, or amino acid mixture on sodium content (mg/100 g dry wt.) of shoot and root of two barley cultivars at the seedling stage.

Treatments	Sodium			
	Shoot		Root	
	G124	G119	G124	G119
Control	18.0±0.0	16.4±0.2	1.23±0.03	0.36±0.03
NaCl	23.3±0.1	17.7±0.3	1.26±0.07	0.68±0.01
Glutathione	15.3±0.1	13.0±0.1	0.71±0.09	0.45±0.06
Glutathione plus salt	15.9±0.1	13.4±0.1	0.67±0.06	0.67±0.01
Ascorbic acid	15.8±0.0	15.6±0.1	0.63±0.03	0.46±0.01
Ascorbic acid plus salt	17.4±0.1	16.2±0.0	0.72±0.10	0.94±0.04
Amino acid mixtures	15.6±0.1	15.3±0.1	0.69±0.07	0.78±0.03
Amino acids plus salt	16.4±0.0	15.9±0.1	0.63±0.03	0.99±0.07
F	834.3	292.9	42.2	87.7
P	**	**	**	**
LSD	0.26	0.27	0.12	0.07

** Highly significant at P≤0.01.

**Table 15.** Effect of 100 mM NaCl with or without glutathione, ascorbic acid, or amino acid mixture on nitrogen content (mg/g dry wt.) of shoot and root of two barley cultivars at the seedling stage.

Treatments	Nitrogen			
	shoot		root	
	G124	G119	G124	G119
Control	0.55±0.0	0.33±0.0	0.25±0.0	0.36±0.0
NaCl	0.02±0.0	0.62±0.0	0.01±0.0	0.36±0.0
Glutathione	0.14±0.0	0.67±0.0	0.71±0.0	0.49±0.0
Glutathione plus salt	0.06±0.0	0.27±0.0	0.47±0.0	0.28±0.0
Ascorbic acid	0.16±0.0	0.22±0.0	0.09±0.0	0.41±0.0
Ascorbic acid plus salt	0.12±0.0	0.76±0.0	0.01±0.0	0.07±0.0
Amino acid mixtures	0.06±0.0	0.34±0.0	0.14±0.0	0.18±0.0
Amino acids plus salt	0.31±0.0	0.33±0.0	0.37±0.0	0.13±0.0
F	988.9	548.6	4247.3	1231.5
P	**	**	**	**
LSD	0.02	0.02	0.01	0.01

** Highly significant at P≤0.01.

**Table 16.** Effect of 100 mM NaCl with or without glutathione, ascorbic acid, or amino acid mixture on potassium/sodium ratio of shoot and root of two barley cultivars at the seedling stage.

Treatments	K/Na ratio			
	shoot		root	
	G124	G119	G124	G119
Control	1.06±0.00	1.04±0.01	0.66±0.06	1.25±0.25
NaCl	0.91±0.01	0.99±0.01	0.62±0.04	0.67±0.07
Glutathione	0.99±0.02	1.04±0.01	0.65±0.09	1.66±0.02
Glutathione plus salt	0.87±0.00	1.22±0.01	0.94±0.05	1.13±0.10
Ascorbic acid	1.06±0.01	0.96±0.01	1.18±0.08	0.99±0.00
Ascorbic acid plus salt	1.12±0.00	0.93±0.01	0.88±0.10	0.48±0.01
Amino acid mixtures	1.10±0.00	1.02±0.00	0.99±0.02	0.83±0.04
Amino acids plus salt	1.27±0.01	0.92±0.01	1.24±0.01	0.74±0.07
F	286.6	201.4	38.1	34.2
P	**	**	**	**
LSD	0.02	0.01	0.11	0.19

** Highly significant at P≤0.01.

**Table 17.** Effect of 100 mM NaCl with or without glutathione, ascorbic acid, or amino acid mixture on protein nitrogen (%) of shoot and root of two barley cultivars at the seedling stage.

Treatments	Protein nitrogen			
	Shoot		Root	
	G124	G119	G124	G119
Control	3.4±0.03	3.9±0.06	1.6±0.03	2.3±0.01
NaCl	2.8±0.04	2.1±0.04	1.7±0.02	2.3±0.03
Glutathione	2.1±0.03	4.2±0.00	4.4±0.05	3.1±0.05
Glutathione plus salt	0.4±0.03	1.7±0.22	2.9±0.05	1.8±0.04
Ascorbic acid	1.0±0.03	2.0±0.02	0.6±0.01	2.6±0.01
Ascorbic acid plus salt	0.8±0.03	4.8±0.03	0.4±0.07	0.4±0.01
Amino acid mixtures	2.0±0.00	2.1±0.03	0.9±0.01	1.2±0.01
Amino acids plus salt	0.4±0.01	2.1±0.10	2.3±0.03	0.8±0.01
F	2519.3	495.5	2850.1	2727.2
P	**	**	**	**
LSD	0.06	0.16	0.07	0.05

** Highly significant at P≤0.01.

Statistical analysis showed that the effects of salinity, glutathione, ascorbic acid, or amino acid mixture with or without salt were highly significant (P≤0.01) in phosphorus, potassium, sodium, K ^+^/Na ^+^ ratio, nitrogen, and protein nitrogen.

8- **Polyamines (PAs) content:** Determination of polyamine content under salt stress indicated an increase in spermidine and spermine in Giza 119 compared to Giza 124 while putrescine increased in both cultivars with salt compared to control ( Table 18). Addition of glutathione with salt caused an increase in putrescine and spermidine of Giza 124 and ascorbic acid with salt resulted in higher putrescine content in each cultivar with an increase in spermidine of Giza 119 only. Also, addition of amino acid mixture with salt showed an increase in putrescine and spermidine in each cultivar. The amount of spermine was so small to be detected with other treatments.

**Table 18.** Effect of 100 mM NaCl with or without glutathione, ascorbic acid, or amino acid mixture on the polyamine content in the two barley cultivars at the seedling stage.

Treatments	Putrescine		Spermidine		Spermine	
	G124	G119	G124	G119	G124	G119
Control	16.1	7.1	1.4	1.11	7.3	2.3
NaCl	15.1	12.2	1.3	2	4.9	6.7
Glutathione	5.1	7.9	2.1	7.1	ND	ND
Glutathione plus salt	5.3	7.6	4	5.2	ND	ND
Ascorbic acid	2.0	2.6	6.3	2.1	ND	ND
Ascorbic acid plus salt	7.3	10.3	2.2	7.4	ND	ND
Amino acid mixtures	ND	ND	ND	ND	ND	ND
Amino acids plus salt	3.8	0.8	1	3.9	ND	ND

### Pre-flowering stage

**Figure 18.**
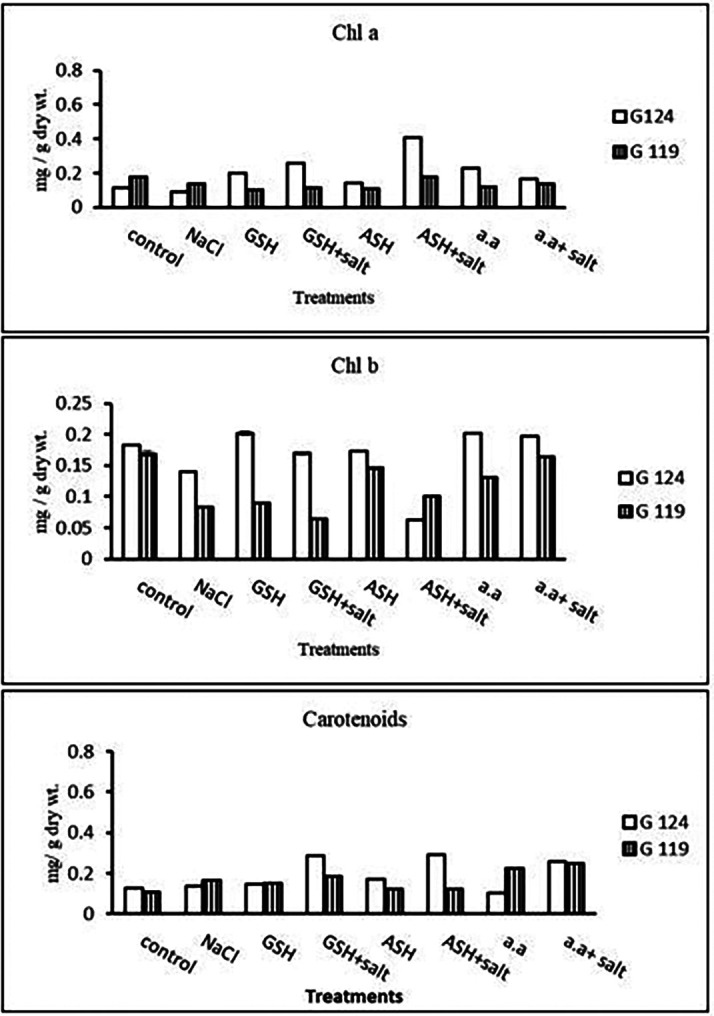
Effect of 100 mM NaCl with or without glutathione, ascorbic acid, or amino acid mixture on photosynthetic pigments (mg/g dry wt.) of the two barley cultivars at the preflowering stage.

**Figure 19.**
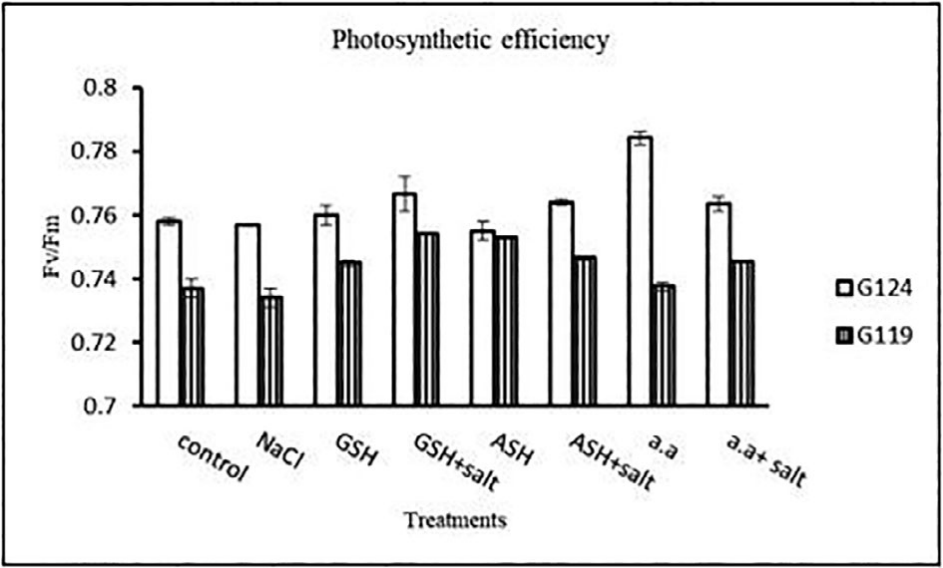
Effect of 100 mM NaCl with or without glutathione, ascorbic acid, or amino acid mixture on photosynthetic efficiency of the two barley cultivars at the preflowering stage.

**Figure 20.**
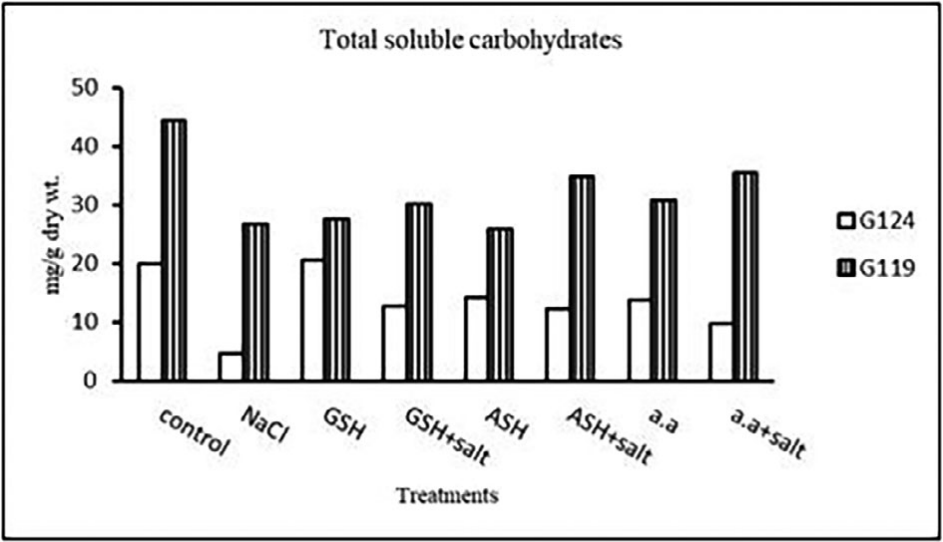
Effect of 100 mM NaCl with or without glutathione, ascorbic acid, or amino acid mixture on the total soluble carbohydrates (mg/g dry wt.) of the two barley cultivars at the preflowering stage.

**Figure 21.**
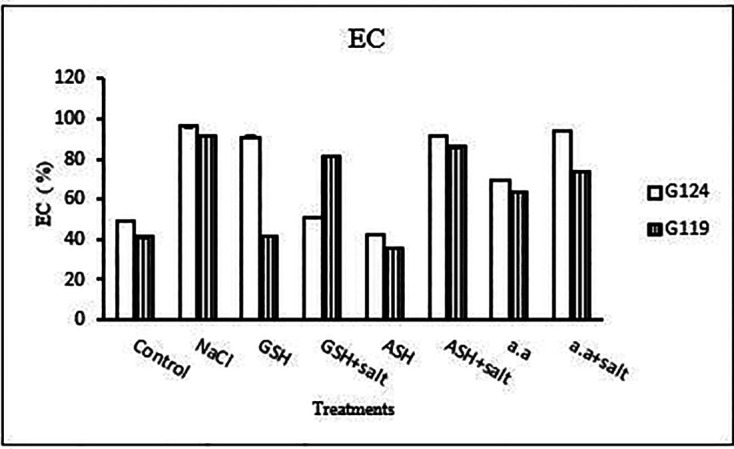
Effect of 100 mM NaCl with or without glutathione, ascorbic acid, or amino acid mixture on membrane leakage (EC %) of the two barley cultivars at the preflowering stage.

1- **Photosynthetic pigments:** In Giza 124 Chl a and Chl b were most dominant in all treatments. A remarkable rise has been observed in Chl b with glutathione, ascorbic acid, or amino acid mixture alone in G124 ( Table 19 and Figure 18). Regarding carotenoids, Giza 124 has higher levels with glutathione plus salt or ascorbic acid plus salt compared to Giza 119.
2- **Photosynthetic efficiency:** Photosynthetic efficiency decreased with salt in each cultivar. In general, Giza 124 showed slightly higher photosynthetic efficiency compared to the Giza119 cultivar, particularly with glutathione or amino acid mixture with or without salt ( Table 20 and Figure 19).
3- **Total Soluble Carbohydrates:** At the pre-flowering stage, salinity showed a decrease in total soluble carbohydrates in each cultivar compared to the control ( Table 21 and Figure 20). By the addition of either glutathione, ascorbic acid or amino acid mixture an increase was detected in total soluble carbohydrates of each cultivar.
4- **Membrane Leakage:** Results showed that membrane leakage was increased under the effect of salinity in each cultivar compared to the control ( Table 22 and Figure 21). Glutathione plus salt showed a decrease in membrane leakage in G124 while Giza119 showed a decrease with amino acid plus salt compared to other treatments.

**Table 19.** Effect of 100 mM NaCl with or without glutathione, ascorbic acid, or amino acid mixture on chlorophyll a, chlorophyll b, and carotenoids of two barley cultivars at the pre-flowering stage.

Treatments	Chlorophyll a		Chlorophyll b		Carotenoids	
	G124	G119	G 124	G119	G 124	G119
Control	0.09±0.0	0.13±0.0	0.14±0.0	0.18±0.0	0.12±0.0	0.16±0.0
NaCl	0.11±0.0	0.18±0.0	0.18±0.0	0.07±0.0	0.13±0.0	0.11±0.0
Glutathione	0.17±0.0	0.10±0.0	0.89±0.0	0.09±0.0	0.15±0.0	0.15±0.0
Glutathione plus salt	0.26±0.0	0.11±0.0	0.17±0.0	0.06±0.0	0.28±0.0	0.18±0.0
Ascorbic acid	0.14±0.0	0.11±0.0	0.23±0.0	0.12±0.0	0.17±0.0	0.12±0.0
Ascorbic acid plus salt	0.39±0.0	0.17±0.0	0.43±0.0	0.29±0.0	0.29±0.0	0.12±0.0
Amino acid mixtures	0.16±0.0	0.12±0.0	0.30±0.0	0.13±0.0	0.10±0.0	0.23±0.0
Amino acids plus salt	0.23±0.0	0.13±0.0	0.17±0.0	0.35±0.0	0.26±0.0	0.25±0.0
F	1680.25	9871.8	22345.1	1636.1	4255.8	22517.2
P	**	**	**	**	**	**
LSD	7.06	3.53	4.99	6.11	3.53	9.99

** Highly significant at P≤0.01.

**Table 20.** Effect of 100 mM NaCl with or without glutathione, ascorbic acid, or amino acid mixture on photosynthetic efficiency of two barley cultivars at the preflowering stage.

Treatments	Photosynthetic efficiency	
	G124	G119
Control	0.76±0.0	0.74±0.0
NaCl	0.75±0.0	0.73±0.0
Glutathione	0.76±0.0	0.75±0.0
Glutathione plus salt	0.77±0.0	0.75±0.0
Ascorbic acid	0.76±0.0	0.75±0.0
Ascorbic acid plus salt	0.76±0.0	0.75±0.0
Amino acid mixtures	0.78±0.0	0.74±0.0
Amino acids plus salt	0.76±0.0	0.75±0.0
F	22.66	45.14
P	**	**
LSD	6.11	3.53

** Highly significant at P≤0.01.

**Table 21.** Effect of 100 mM NaCl with or without glutathione, ascorbic acid, or amino acid mixture on total soluble carbohydrates (mg/g dry wt.) of shoot of two barley cultivars at the preflowering stage.

Treatments	Total soluble carbohydrates	
	G124	G119
Control	19.8±0.0	44.4±0.2
NaCl	4.5±0.2	26.7±0.2
Glutathione	20.6±0.1	27.6±0.2
Glutathione plus salt	12.7±0.2	30.1±0.0
Ascorbic acid	14.1±0.1	25.8±0.2
Ascorbic acid plus salt	12.4±0.1	34.7±0.1
Amino acid mixtures	13.8±0.1	30.8±0.0
Amino acids plus salt	9.7±0.0	35.4±0.0
F	4435.7	9681.6
P	**	**
LSD	0.34	0.18

** Highly significant at P≤0.01.

**Table 22.** Effect of 100 mM NaCl with or without glutathione, ascorbic acid, or amino acid mixture on membrane leakage (EC %) of two barley cultivars at the pre-flowering stage.

Treatments	Membrane leakage	
	G124	G119
Control	49.1±0.2	41.2±0.8
NaCl	96.2±0.6	91.2±0.4
Glutathione	90.9±0.6	41.8±0.1
Glutathione plus salt	50.7±0.3	81.6±0.2
Ascorbic acid	42.5±0.4	35.8±0.1
Ascorbic acid plus salt	91.6±0.2	86.0±1.8
Amino acid mixtures	69.2±0.3	63.5±0.3
Amino acids plus salt	94.3±1.1	73.7±0.1
F	11085.3	6402.9
P	**	**
LSD	0.65	0.83

** Highly significant at P≤0.01.

Statistical analysis showed that the effects of salinity, glutathione, ascorbic acid or amino acid mixture with or without salt were highly significant (P≤0.01) on photosynthetic pigments, photosynthetic efficiency, total soluble carbohydrates, and membrane leakage.

### Yield measurements

**Figure 22.**
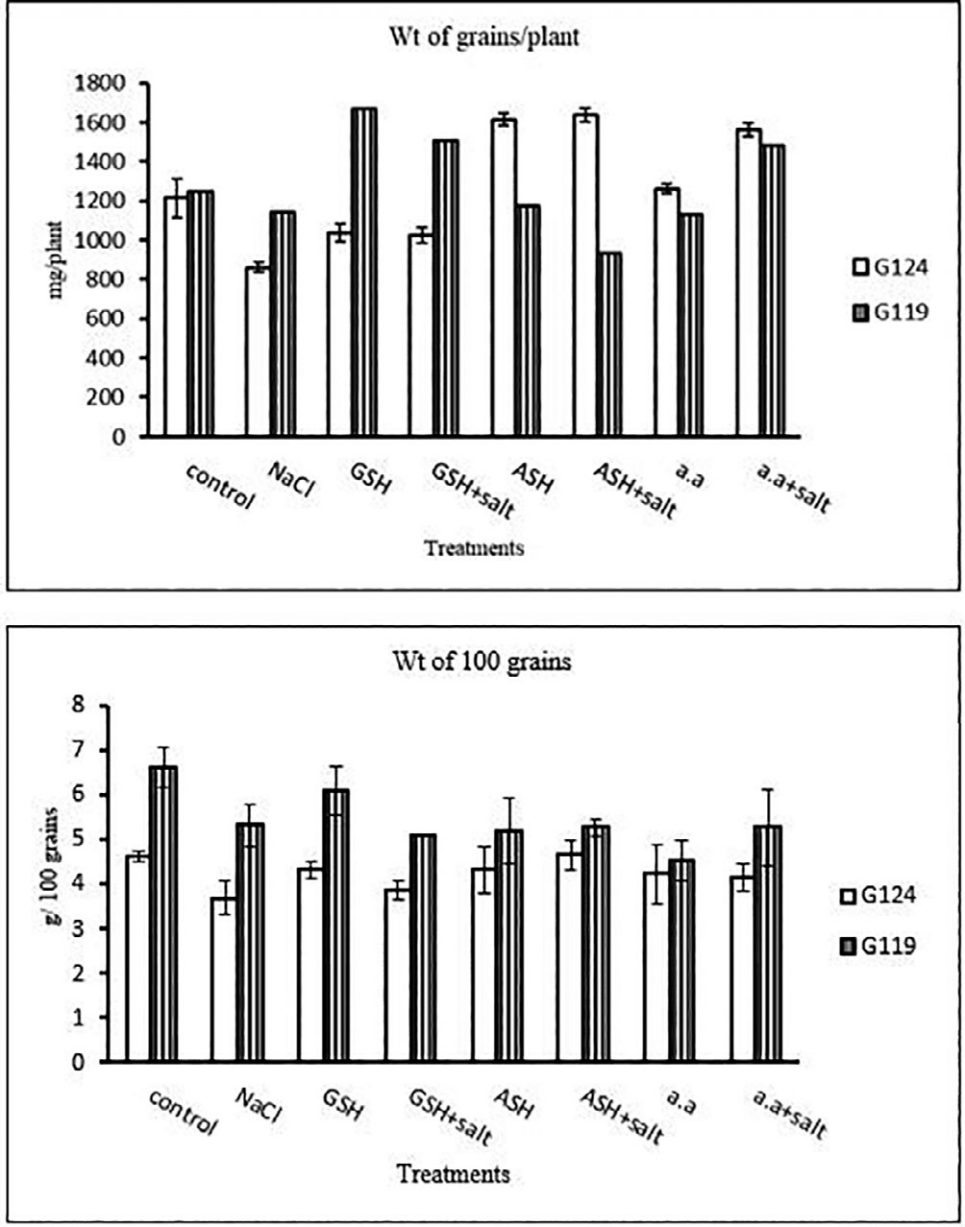
Effect of 100 mM NaCl with or without glutathione, ascorbic acid, or amino acid mixture on weight of grains (mg/plant) and weight of 100 grain (g/100 grains) of each cultivar.

**Figure 23.**
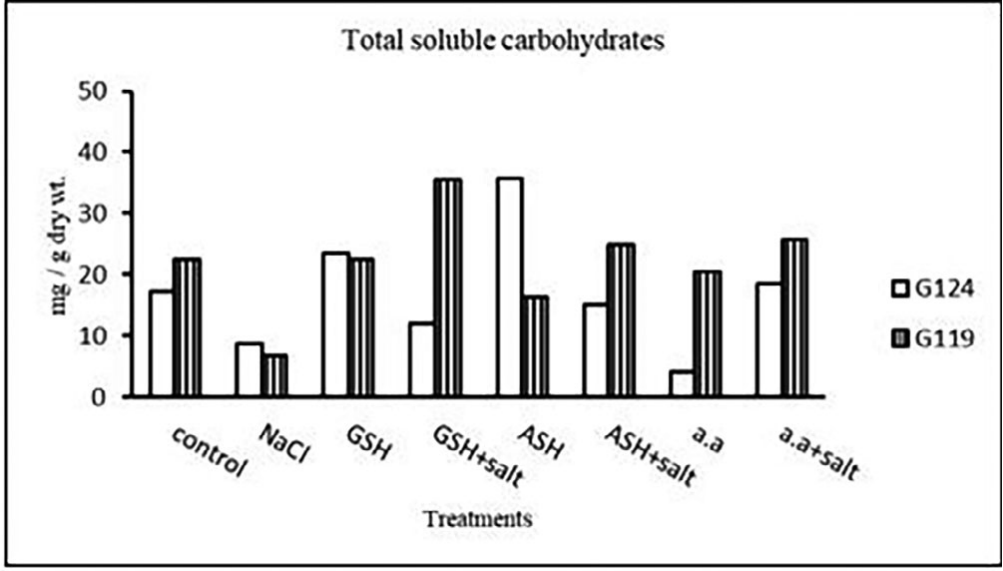
Effect of 100 mM NaCl with or without glutathione, ascorbic acid, or amino acid mixture on total soluble carbohydrates (mg/g dry wt.) of grains of the two barley cultivars.

**Figure 24.**
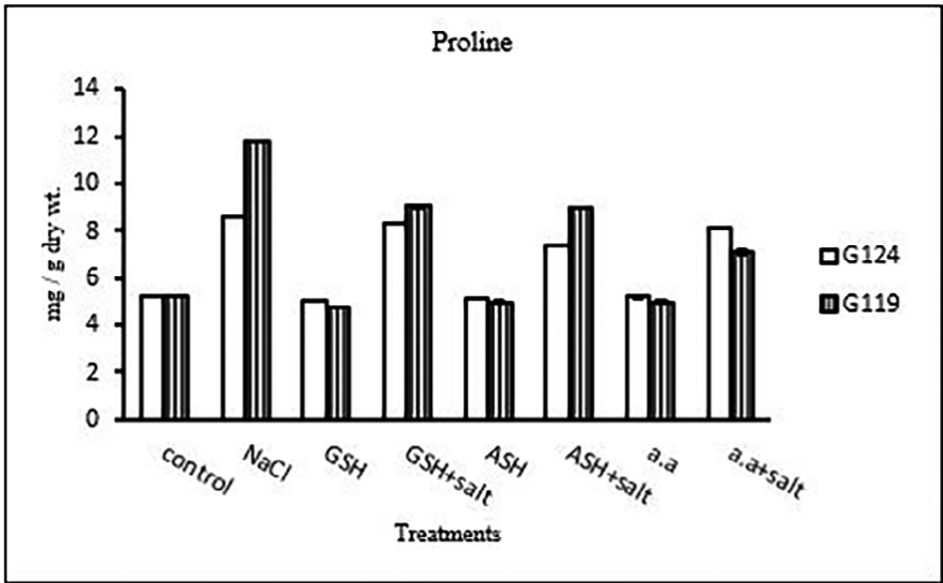
Effect of 100 mM NaCl with or without glutathione, ascorbic acid, or amino acid mixture on proline content (mg/g dry wt.) of grains of the two barley cultivars.

**Figure 25.**
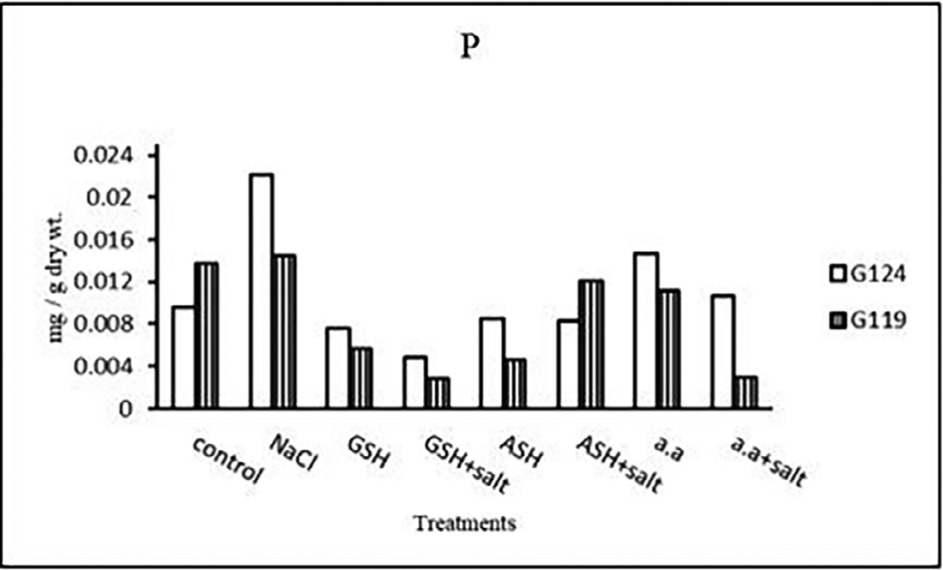
Effect of 100 mM NaCl with or without glutathione, ascorbic acid or amino acid mixture on phosphorous content (mg/g dry wt.) of grains of the two barley cultivar.

**Figure 26.**
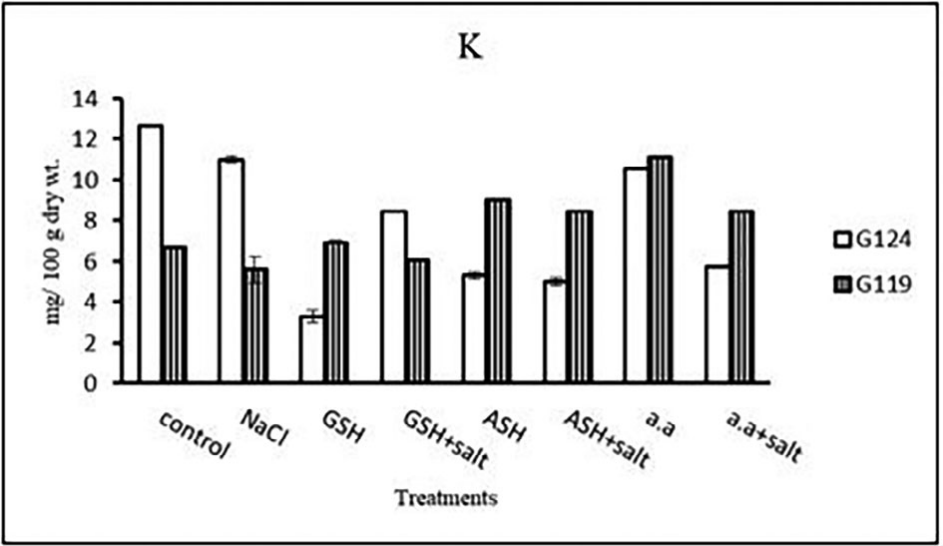
Effect of 100 mM NaCl with or without glutathione, ascorbic acid or amino acid mixture on potassium content (mg/100 g dry wt.) of grains of the two barley cultivars.

**Figure 27.**
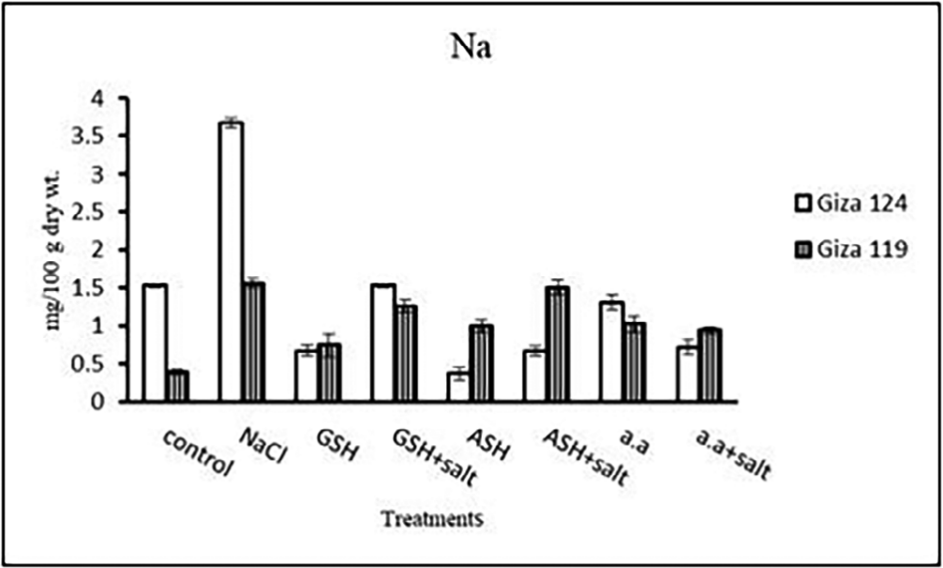
Effect of 100 mM NaCl with or without glutathione, ascorbic acid or amino acid mixture on sodium content (mg/100 g dry wt.) of grains of the two barley cultivars.

**Figure 28.**
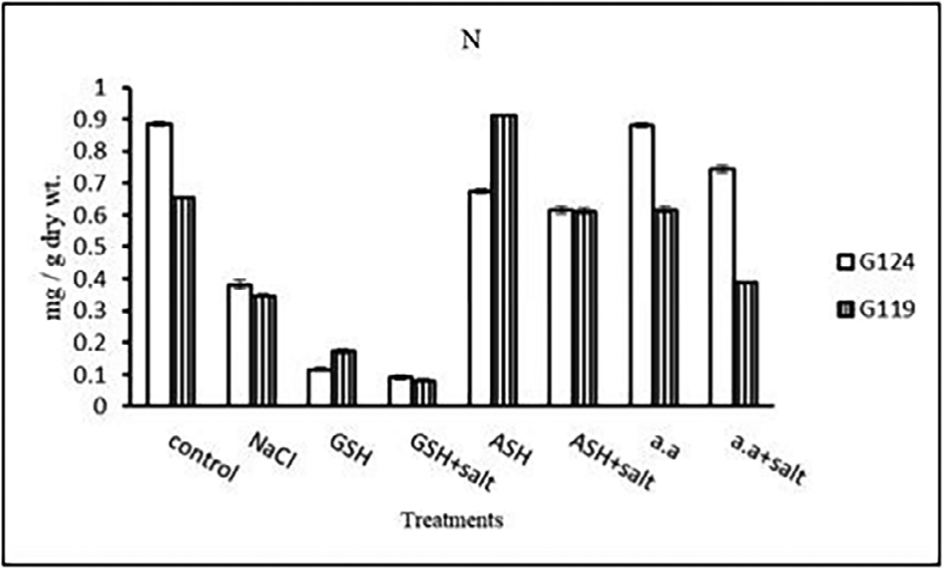
Effect of 100 mM NaCl with or without glutathione, ascorbic acid or amino acid mixture on nitrogen content (mg/g dry wt.) of grains of the two barley cultivars.

**Figure 29.**
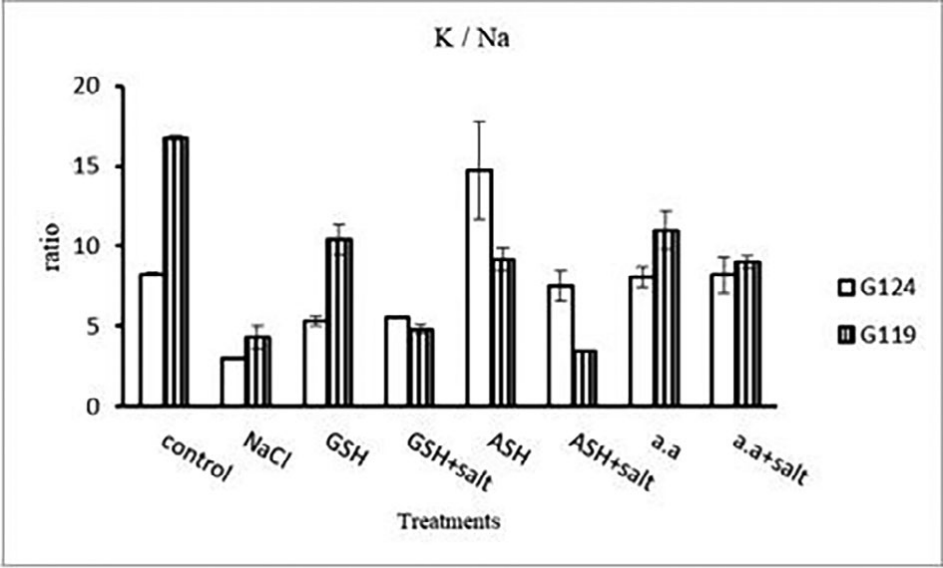
Effect of 100 mM NaCl with or without glutathione, ascorbic acid or amino acid mixture on K ^+^/Na ^+^ ratio of grains of the two barley cultivars.

**Figure 30.**
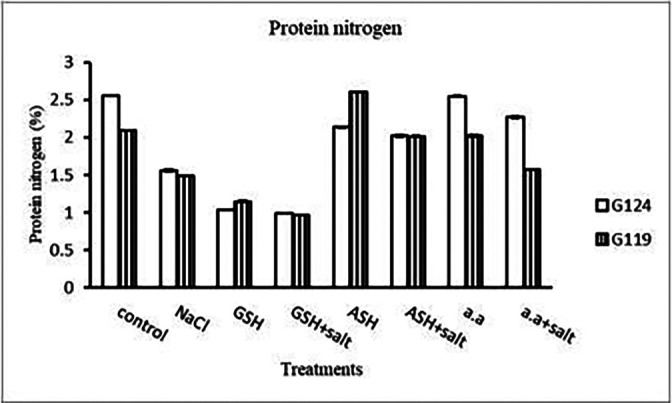
Effect of 100 mM NaCl with or without glutathione, ascorbic acid or amino acid mixture on protein nitrogen (%) of grains of the two barley cultivars.

1- **Yield criteria:** In both cultivars weight of grains per plant and weight of 100 grains has decreased under the effect of salinity compared to the control ( Table 23 and Figure 22). Grains per plant were increased in Giza 124 with either ascorbic acid or amino acid mixture and in Giza 119 with glutathione alone or plus salt and amino acid plus salt in Giza 119.
2- **Total soluble carbohydrates:** It can be noticed ( Table 24 and Figure 23) that salinity brought about a drop in total soluble carbohydrates in each cultivar but increased in both with glutathione alone. The addition of glutathione, ascorbic acid or amino acid mixture to salt has resulted in a rise of T.S.C in Giza 119-seeds compared to that of Giza 124.
3- **Proline content:** In both cultivars, a specific increase in grain proline has been seen when sodium chloride, glutathione, ascorbic acid, or an amino acid mixture are combined with salt in comparison to control ( Table 25, and Figure 24). According to statistical analysis, each cultivar's proline content, total soluble carbohydrates, and yield criteria were all significantly affected by salinity, glutathione, ascorbic acid, or amino acid mixtures with or without salt (P≤0.01).
4- **Mineral analysis**
  a) **Phosphorous content:** In each cultivar, phosphorus content was increased under salinity compared to the control ( Table 26 and Figure 25). Phosphorus content increased in Giza 124 with amino acid mixture alone compared to 119, while increased in Giza 119 with ascorbic acid plus salt.
  b) **Potassium content:** In both cultivars, potassium content showed a low level under salinity compared to control ( Table 27 and Figure 26). On the other hand, potassium content increased in Giza 119 with glutathione, ascorbic acid or amino acid mixture with or without salt.
  c) **Sodium content:** An apparent rise has been detected in Na content of Giza 124 and Giza 119. Other treatments showed a decrease in sodium content in each cultivar ( Table 28 and Figure 27). K ^+^/Na ^+^ ratio decreased under salinity in each cultivar, while it increased with ascorbic acid alone in G124 and with glutathione, ascorbic acid or amino acid mixture without salt in G119 ( Table 30 and Figure 29).
  d) **Nitrogen content:** In both cultivars, nitrogen content showed a remarkable reduction with salinity ( Table 29 and Figure 28). However, the highest content was observed with the amino acid mixture in Giza 124 and ascorbic acid in Giza 119, while the lowest one was observed in both cultivars with glutathione plus salt.
  e) **Protein nitrogen:** In comparison to other treatments, protein nitrogen decreased with salt in both cultivars and noticeably with glutathione in two cultivars, either with or without salt ( Table 31 and Figure 30). With ascorbic acid or an amino acid and salt, protein nitrogen increased in both cultivars.

**Table 23.** Effect of 100 mM NaCl with or without glutathione, ascorbic acid, or amino acid mixture on weight of grains per plant (mg/plant) and weight of 100 grains (g/100 grains) of grains in the two barley cultivars.

Treatments	Weight of grains/plant		Weight of 100 grians	
	G124	G119	G124	G119
Control	1213.3±101.2	1248.3±5.77	4.62±0.1	6.62±0.4
NaCl	860.0±26.5	1141.7±7.63	3.70±0.4	5.33± 0.4
Glutathione	1038.3±46.5	1668.3±88.1	4.33±0.2	6.12± 0.4
Glutathione plus salt	1026.7±40.1	1508.3±7.63	3.87±0.2	5.12±0.0
Ascorbic acid	1613.3±30.6	1175.0±26.5	4.33± 0.5	5.20± 0.7
Ascorbic acid plus salt	1636.7± 32.1	931.7±40.1	4.66± 0.3	5.29±0.2
Amino acid mixtures	1261.7±25.2	1130.0±15.0	4.25± 0.7	4.54±0.4
Amino acids plus salt	1561.7±33.3	1485.0±70.0	4.17±0.3	5.29±0.9
F	115.72	93.78	2.36	4.50
P	**	**	ns	**
LSD	83.03	75.88	0.64	0.91

** Highly significant at P≤0.01.

**Table 24.** Effect of 100 mM NaCl with or without glutathione, ascorbic acid, or amino acid mixture on total soluble carbohydrates (mg/g dry wt.) of grains in the two barley cultivars.

Treatments	Total soluble carbohydrates	
	G124	G119
Control	17.3±0.1	22.5±0.1
NaCl	8.6±0.0	6.7±0.0
Glutathione	23.6±0.0	22.4±0.0
Glutathione plus salt	11.9±0.1	35.4±0.0
Ascorbic acid	35.7±0.3	16.4±0.0
Ascorbic acid plus salt	15.1±0.0	24.7±0.1
Amino acid mixtures	4.0±0.0	20.4±0.0
Amino acids plus salt	18.5±0.1	25.8±0.1
F	21566.9	86611.5
P	**	**
LSD	0.20	0.08

ns=not significant.

** Highly significant at P≤0.01.

**Table 25.** Effect of 100 mM NaCl with or without glutathione, ascorbic acid, or amino acid mixture on proline content (mg/g dry wt.) of grains in the two barley cultivars.

Treatments	Proline	
	G124	G119
Control	5.2±0.1	5.2±0.1
NaCl	8.6±0.0	11.8±0.0
Glutathione	5.1±0.0	4.8±0.0
Glutathione plus salt	8.4±0.0	9.0±0.1
Ascorbic acid	5.1±0.0	4.9±0.1
Ascorbic acid plus salt	7.4±0.1	8.9±0.0
Amino acid mixtures	5.2±0.1	4.9±0.1
Amino acids plus salt	8.2±0.1	7.1±0.1
F	2266.13	2792.5
P	**	**
LSD	0.10	0.14

** Highly significant at P≤0.01.

**Table 26.** Effect of 100 mM NaCl with or without glutathione, ascorbic acid, or amino acid mixture on the phosphorus content (mg/g dry wt.) of grains in the two barley cultivars.

Treatments	Phosphorus	
	G124	G119
Control	0.009±0.0	0.013±0.0
NaCl	0.022±0.0	0.014±0.0
Glutathione	0.007±0.0	0.006±0.0
Glutathione plus salt	0.004±0.0	0.002±0.0
Ascorbic acid	0.008±0.0	0.004±0.0
Ascorbic acid plus salt	0.008±0.0	0.012±0.0
Amino acid mixtures	0.014±0.0	0.011±0.0
Amino acids plus salt	0.010±0.0	0.002±0.0
F	35099.2	24607.0
P	**	**
LSD	8.65	9.34

** Highly significant at P≤0.01.

**Table 27.** Effect of 100 mM NaCl with or without glutathione, ascorbic acid, or amino acid mixture on potassium content (mg/100 g dry wt.) of grains in the two barley cultivars.

Treatments	Potassium	
	G124	G119
Control	12.6± 0.0	6.6± 0.1
NaCl	11.0± 0.1	5.6± 0.1
Glutathione	3.3± 0.3	6.9± 0.1
Glutathione plus salt	8.5± 0.1	6.0± 0.0
Ascorbic acid	5.3± 0.2	9.0± 0.0
Ascorbic acid plus salt	5.0± 0.2	8.4± 0.4
Amino acid mixtures	10.5±0.0	11.1± 0.0
Amino acids plus salt	5.8±0.1	8.5± 0.1
F	1516.8	10674.2
P	**	**
LSD	0.26	0.05

** Highly significant at P≤0.01.

**Table 28.** Effect of 100 mM NaCl with or without glutathione, ascorbic acid or amino acid mixture on sodium content (mg/100 g dry wt.) of grains in the two barley cultivars.

Treatments	Sodium	
	G124	G119
Control	1.53±0.03	0.39±0.07
NaCl	3.67± 0.06	1.56±0.07
Glutathione	0.67± 0.07	0.60±0.11
Glutathione plus salt	1.53±0.03	1.26±0.07
Ascorbic acid	0.37± 0.07	0.99±0.07
Ascorbic acid plus salt	0.67± 0.06	2.44±0.04
Amino acid mixtures	1.31± 0.09	1.02± 0.10
Amino acids plus salt	0.71± 0.09	0.94±0.04
F	1327.4	282.4
P	**	**
LSD	0.08	0.11

** Highly significant at P≤0.01.

**Table 29.** Effect of 100 mM NaCl with or without glutathione, ascorbic acid or amino acid mixture on nitrogen content (mg/g dry wt.) of grains in the two barley cultivars.

Treatments	Nitrogen	
	G124	G119
Control	0.88±0.007	0.65±0.003
NaCl	0.38±0.012	0.35±0.006
Glutathione	0.12±0.004	0.17±0.008
Glutathione plus salt	0.11±0.006	0.08±0.004
Ascorbic acid	0.67±0.009	0.91±0.001
Ascorbic acid plus salt	0.62±0.011	0.61±0.011
Amino acid mixtures	0.88±0.007	0.61±0.009
Amino acids plus salt	0.74±0.012	0.39±0.004
F	2802.8	4233.8
P	**	**
LSD	0.02	0.01

** Highly significant at P≤0.01.

**Table 30.** Effect of 100 mM NaCl with or without glutathione, ascorbic acid or amino acid mixture on potassium/sodium ratio of grains in the two barley cultivars.

Treatments	Potassium/sodium	
	G124	G119
Control	8.2±0.1	16.7±0.1
NaCl	2.9±0.1	4.2±3.7
Glutathione	5.3±0.5	7.4±5.6
Glutathione plus salt	5.5±0.1	4.8±0.2
Ascorbic acid	14.7±3.0	9.2±0.7
Ascorbic acid plus salt	7.5±0.9	3.5±0.1
Amino acid mixtures	8.0±0.6	10.9±1.2
Amino acids plus salt	8.2±1.1	9.0±0.4
F	23.4	27.0
P	**	**
LSD	2.13	2.53

** Highly significant at P≤0.01.

**Table 31.** Effect of 100 mM NaCl with or without glutathione, ascorbic acid or amino acid mixture on protein nitrogen (%) of grains in the two barley cultivars.

Treatments	Protein nitrogen	
	G124	G119
Control	2.5±0.01	2.1±0.01
NaCl	1.5±0.01	1.4±0.01
Glutathione	1.0±0.01	1.1±0.02
Glutathione plus salt	0.9±0.01	0.9±0.01
Ascorbic acid	2.1±0.01	2.6±0.00
Ascorbic acid plus salt	2.0±0.02	2.0±0.02
Amino acid mixtures	2.5±0.01	2.0±0.01
Amino acids plus salt	2.7±0.02	1.5±0.01
F	3315.4	4650.1
P	**	**
LSD	0.03	0.02

** Highly significant at P≤0.01.

According to statistical analysis, salinity, glutathione, ascorbic acid, or an amino acid mixture with or without salt had a highly significant impact (P≤0.01) on the levels of phosphorus, potassium, sodium, the K/Na ratio, nitrogen, and protein nitrogen.

## Discussion

In the present study, salt stress significantly decreased all growth parameters, including fresh weight, dry weight, shoot height, root depth, succulence, and leaf area in both salt-tolerant and salt-sensitive barley cultivars. The reduction in leaf area under salt stress means that photosynthesis is decreased ( Abdel-Farid *et al.*, 2020; Munns & Tester, 2008). Such decrease may be partly due to decreased cell water potential associated with stomatal closure thereby, decreased CO _2_ assimilation, besides NaCl toxicity and decreased availability of water. These results were in agreement with ( Razzaque *et al.*, 2009).

The current findings supported those of ( Asch *et al.*, 2000) who studied various rice cultivars and found that the salt-tolerant cultivar (Giza 124) showed less dry matter degradation than the sensitive one. As anti-oxidant compounds, glutathione (GSH) and ascorbic acid (ASH) were found to be effective in raising all growth parameters, which may be related to their inhibitory effect on the uptake of Cl- and Na+ ions and their stimulatory effect on the uptake of the necessary elements, such as N, Mg, and Fe ( Abd El-Hameid & Sadak, 2020; Munir *et al.*, 2021). ( Rawia *et al.*, 2011) reported that exogenous application of glutathione or ascorbic acid mitigated partially or completely the adverse effects of salt stress on the growth of canola seedlings and Marigold plants, respectively. In this study, the addition of a mixture containing a number of amino acids; arginine, ornithine and methionine has induced an increase in the shoot and root dry weights of salt stressed seedlings of each cultivar. In this regard ( El-Shourbagy, 1964), showed a similar effect of amino acid mixture with the *in vitro* culture of tomato roots. However ( Abo Sedera *et al.*, 2010; El-Zohiri & Asfour, 2009), showed that the effect of polyamine precursors on the protein assimilation and cell formation of some potato cultivars can be attributed to the assimilation of gibberellins biosynthesis.

Salt stress induced a significant reduction in chlorophyll a and b levels in the two cultivars of barley which agreed with the results of ( Taïbi *et al.*, 2016) for two genotypes, high-yielding ‘Tema’ and low-yielding ‘Djadida’, commonly cultivated in Algeria. This decrease may be due to increased chlorophyllase activity as reported by ( Nazarbeygi *et al.*, 2011) and also may be caused by suppression of specific enzymes responsible for the synthesis of green pigments ( Kaur *et al.*, 2014; Mishra *et al.*, 2006). In contrast, carotenoids were significantly increased in the salt stressed barley cultivars and this may be due to their role in the protection of the photosystems by reacting with the lipid peroxidation products ( Borghesi *et al.*, 2011).

In Giza 124, glutathione and ascorbic acid with or without salt significantly increased chlorophyll a levels under salt stress compared to the control. Amini and Ehsanpour (2005) found similar results with canola seeds. In comparison to the control, G119 showed an increase in carotenoids when treated with glutathione, ascorbic acid, or an amino acid combination with or without salt. Glutathione's impacts on pigment levels may be due to their effects on either enhancing photosynthetic activities and chlorophyll biosynthesis or retarding chlorophyll degradation caused by oxidative stress. The addition of amino acids (polyamine precursors) has, on the other hand, resulted in an increase in chl b in both cultivars. Similar results have been reached with garlic plants ( El-Shabasi *et al.*, 2005), and sweet pepper ( Al-Said & Kamal, 2008). This increase may be due to the role of amino acid mixture in detoxification of harmful accumulated of ROS in the thylakoid membrane during photosynthesis.

The current study showed a highly significant decrease in photosynthetic efficiency (Fv/Fm) under salt stress, which can be attributed to photochemical quenching in PSII. Salt stress increased reactive oxygen species formation, which damaged PSII and organelles such as chloroplasts, mitochondria, and plasma membranes. As a result, PSII's efficiency decreases. Several authors have reported comparable outcomes with several crop species under salt stress ( Alnusairi *et al.*, 2021). When glutathione, ascorbic acid, or an amino acid combination (polyamine precursors) were combined with salt, photosynthetic efficiency increased compared to the control. This could be related to their role in mitigating the harmful effects of salt via ROS scavenging.

There was a highly significant decrease in the total soluble carbohydrates in shoot and root of each cultivar under salinity stress compared with the control at the three studied growth stages. However, the salt-tolerant cultivar (Giza 124) has generally greater total soluble sugars compared to the salt-sensitive one (Giza 119) and this agreed with the results obtained by Almodares *et al.* (2008) and Ashraf and Tufail (1995) in sunflower. In each barley cultivar, the decrease in the total soluble carbohydrates under salt stress may be due to that the building up in the chloroplast extent a direct toxic effect on photosynthetic processes ( Munns & Tester, 2008) or to a reduction in CO _2_ assimilation rate and in the stomatal conductance. However, treatment with GSH or ASH has stimulated the accumulation of total soluble carbohydrates which are required as compatible solutes for osmoregulation in plants under water and salt-stresses ( Hare *et al.*, 1998). Accumulation of these compatible solutes reduces the osmotic potential in the cytoplasm and contributes to maintaining water homeostasis among several cellular compartments ( Sairam & Tyagi, 2004), thus tuning the rate of photosynthesis to match the demand arising from grown inhibition.

The effects of glutathione or ascorbic acid on the accumulation of total soluble sugars can probably be attributed to their protective effects on the photosynthetic systems. However, GSH plays a protective role in salinity tolerance through the maintenance of the redox status ( Chaparzadeh *et al.*, 2004). Proline, an osmoprotectant, has been shown to accumulate more in plants in response to salinity ( Ueda *et al.*, 2007). Increased cellular proline levels are strongly correlated with the ability to withstand environmental challenges, such as salinity, and they can also act as an organic nitrogen reserve ( Sairam & Tyagi, 2004). Proline levels rose in all barley cultivars when exposed to salinity ( Somayeh *et al.*, 2012), but it accumulated more in the salt-tolerant variety (Giza 124), which is related to its capacity to withstand salinity stress. The higher level of proline content in the two cultivars under salinity may be due to the expression of a gene encoding key enzymes of proline synthesis and low activity of the oxidizing enzymes which is controlled by either osmotic or salinity stress ( Ma *et al.*, 2015; Somayeh *et al.*, 2012). Proline can act as an enzyme protector or a free radical scavenger. Results revealed that adding glutathione, ascorbic acid, or an amino acid combination reduced the level of proline. Such inverse correlation between these antioxidant compounds and proline in barley cultivars can be due to a deficit of common precursors which agreed with the results of Hoque *et al.* (2007) and Okuma *et al.* (2002) on *Geum urbanum.*

However, the tolerant cultivar was found to have larger levels of the ROS-scavenging enzymes catalase and peroxidase than the sensitive one, indicating that the antioxidant system is crucial for plant tolerance against environmental stresses. In this respect ( Nawaz & Ashraf, 2007; Sairam *et al.*, 2000), found that variations in the antioxidant systems of wheat and maize genotypes lead to differences in how the plants react to diverse stresses. According to the current findings, salt stress boosted the CAT and POD activities in both barley varieties. The salt-tolerant (Giza 124) showed a greater increase than the salt-sensitive one, showing that the former was a more effective scavenger for the H _2_O _2_ radical and provided superior protection against H _2_O _2_. Similar results have been observed with *Beta maritime* (halophyte) compared to the non-halophyte *Beta vulgaris* ( Bor *et al.*, 2003) *,* and wheat ( Sairam *et al.*, 2002) differing in salt tolerance. However, the addition of glutathione, ascorbic acid, or an amino acid mixture to salt-treated barley cultivars significantly reduced the activities of catalase and peroxidase, indicating a compensatory or substituting effect for the already present antioxidant enzymes by directly scavenging H _2_O _2_ and other ROS.

Malonyldialdehyde (MDA), a criterion for evaluating salt injury, causes lipid peroxidation in a variety of plants ( Ma *et al.*, 2015; Somayeh *et al.*, 2012). In response to salinity, both barley cultivars showed a highly significant increase in lipid peroxidation (at the seedling stage) and membrane leakage (at the seedling and Pre-flowering stages), with the salt-sensitive cultivar showing these effects more obvious than the salt-tolerant cultivar. The increase in MDA level when exposed to salt may be caused by oxidative damage to the chloroplasts and mitochondria or by antioxidants' inability to completely neutralize and scavenge all of the active oxygen species produced when exposed to salt stress. The present results agreed with those of ( Demiral & Türkan, 2005) for two rice cultivars, ( Chaparzadeh *et al.*, 2004) for *Calendula officinalis.* Increasing lipid peroxidation during salt stress has been reported also ( Xing *et al.*, 2019) with *Portulaca oleracea* L.

In the two barley cultivars, MDA, as a product of the lipid peroxidation, has decreased with the addition of glutathione, ascorbic acid or amino acid mixture due to their compensating effects on the activities of antioxidant enzymes, acting as scavengers of cytotoxic H _2_O _2_ and reacting non-enzymatically with other ROS ( Sairam *et al.*, 2002). The amino acid mixture has reduced MDA content and membrane leakage which agreed with the results of Zhang and Kirkham (1996) for sorghum and sunflower seedlings.

Several authors have reported increased levels of PAs when plants are exposed to diverse kinds of environmental stress ( Bouchereau *et al.*, 1999; Nayyar & Chander, 2004). The present results indicated that under salt stress, PUT increased in the tolerant cultivar (Giza 124) whereas Spd and Spm increased in the sensitive cultivar. In contrast to these findings, ( Krishnamurthy & Bhagwat, 1989) found that during salt stress, rice cultivars that were salt-resistant acquired more Spd and Spm than sensitive ones. Additionally, they noted that sensitive rice accumulated more Put under salt than tolerant rice ( Erdei *et al.*, 1996) indicate that Spd and Spm accumulation in sorghum is one of the adaptive responses to salt stress. ( Chattopadhayay *et al.*, 2002; Willadino *et al.*, 1996) proposed that accumulation of Spd and Spm may contribute to stress tolerance, while PUT accumulation may have no positive effect under salinity stress. Besides, it has been reported that, mainly in cereals, Put accumulation seemed to be related especially to the activation of arginine decarboxylase under stress conditions ( Bouchereau *et al.*, 1999).

Salt stress has increased phosphorus concentration in the shoot and root of each barley cultivar which agreed with the results of ( Taban, 2000; Yahya, 1998). According to ( Roberts *et al.*, 1984) the increase of P level in maize is the result of enhanced rates of uptake by the roots and of translocation to the shoots and not a concentration effect due to growth depression or may be due to ion imbalance between the uptake of sodium and ions including phosphorus ( Lutts *et al.*, 1999). However, under the effect of glutathione, ascorbic acid, or amino acid mixture with or without salt, phosphorus content decreased in each cultivar. This decrease may be due to the ameliorating effect or controlling the uptake of phosphorus which is linked with pH.

Results showed that Giza 124 showed a greater accumulation of sodium under salinity compared to Giza 119, and this agreed with the results of ( Flowers, 2004; He & Cramer, 1992). They found that maize shoots of Giza 2 (salt-tolerant) have greater levels of Na ^+^ compared to those of Tri-hybrid 321(salt sensitive) under NaCl treatments. Furthermore, the greatest accumulation of sodium by plants at high salt concentrations may be attributed to the fact that selective salt absorption may be replaced by passive absorption which causes abnormal accumulation of salts in plant organs ( Bouchereau *et al.*, 1999). They suggested that under salinity sodium influx across the plasmalemma to the vacuole might play a major role in permitting turgor maintenance.

It may be suggested that the cultivar Giza 124 had the ability to sequester Na ^+^ into the vacuole more efficiently than Giza 119. Several reports indicated that the absence of ion compartmentation may contribute to the toxic effects of ions in the shoot of sensitive plants ( Flowers & Yeo, 1997). However, K ^+^ concentration and consequently K ^+^/Na ^+^ ratio were decreased under salt-stress in the leaves of each cultivar. These decreases could be due to the effect of Na ^+^ on K ^+^ transport into the xylem or an inhibition of uptake processes ( Gu *et al.*, 2016; Yan *et al.*, 2013). Again, decreasing in potassium content can be due to an antagonistic effect between sodium and potassium which has been confirmed by Azevedo Neto and Tabosa (2000). These antagonistic relations between Na ^+^ and K ^+^ may be taken as an indication of the role played by hormones in modifying K ^+^/Na ^+^ selectively under salt stress ( Alpaslan & Gunes, 2001). Studies with the two barley cultivars showed that salinity has a significant reducing effect on the K ^+^ concentration in both shoot and root, which can be referred to the competition and resultant selective uptake between K ^+^ and Na ^+^ which causes an increase in uptake of Na ^+^ at the cost of K ^+^ ( Kuiper, 1984) or due to a decrease in sink size with the higher concentrations of NaCl, which strongly inhibited shoot and root growth ( Ashrafuzzaman *et al.*, 2000). Gebauer *et al.* (2003) reported that K ^+^ accumulation was reduced in the stem and increased in leaves and roots with increasing concentration of NaCl. On the other hand ( Flowers & Yeo, 1997). Epstein (1998) claimed that enhanced K ^+^ uptake can be an adaptive mechanism that allows the cell to evade K ^+^ starvation in the presence of high NaCl concentrations, which does not agree with the present study.

Results showed an increase in shoot-nitrogen content in each barley cultivar. Flanagan and Jefferies (1988) showed that when plants are subjected to high salinity, higher nitrogen content was associated with osmotic solute or an accumulation of nitrate ions or increased protein degradation. Application of glutathione, ascorbic acid or amino acid mixture with or without salt has led to a reduction in nitrogen content in the shoot of Giza 124 compared to the control and the treatment of the salt alone, while in Giza 119, glutathione without salt and ascorbic acid with salt caused a higher nitrogen content compared to control and salt alone. This difference can be due to impairment of nitrogen metabolism in the sensitive cultivars while the tolerant one was able to use nitrogen for enzymatic defense system against salt. In contrast, root-nitrogen content decreased with salinity in each cultivar.

Concerning the yield, the present results showed that, in both cultivars, NaCl stress resulted in a reduction in grain weight in both cultivars which can be confirmed with the results of Sairam *et al.* (2000) and Sohrabi *et al.* (2008) for chickpea and ( Taffouo *et al.*, 2009) for some cowpea ( *Vigna unguiculata).* Grain weight reduction can be related to the disturbance in the translocation due to toxic ions or a reduction in the photosynthesis, imbalance in mineral uptake, protein synthesis or carbohydrate metabolism ( Al-Garni, 2006). However ( Zeng *et al.*, 2002, p. 2), reported that the few differences in grain weight of rice ( *Oryza sativa* L.) genotypes under salinity stress can be related to plant genus and genotype.

## Conclusion

Salinity stress resulted in the reduction of all growth parameters, pigment contents, photosynthetic efficiency, total soluble carbohydrates and grain weight in the seedlings, pre-flowering, and grain yield stages. On the other hand, salinity enhanced the activities of the antioxidant enzymes, catalase, and peroxidase, and increased the proline content of the three growth stages. The addition of glutathione, ascorbic acid, or amino acid mixture led to the enhancement of the defense mechanism of barley against salinity stress which was reflected in all previous parameters. Also, the present study indicated that Giza 124 as a salt-tolerant cultivar was more resistant to the damage caused by salt stress compared to Giza 119 as a salt-sensitive one. This salt-sensitive cultivar showed more MDA as a product of lipid peroxidation and membrane leakage with salt stress. The results indicated that the alleviation effect was finally reflected in the growth criteria of the plants suggesting that glutathione may be recommended as the best treatment to alleviate the effect of salinity on barley.

## Data Availability

Figshare: Spreadsheets, https://doi.org/10.6084/m9.figshare.22082354.v1 ( El Dakkak, 2023). This project contains the following underlying data:
- Catalse (seedling).csv - Chlorophyll (preflowering G119).csv - Chlorophyll (preflowering).csv - Chlorophyll-Seedling Stage G119.csv - Chlorophyll-Seedling Stage-G124.csv - Growth Criteria Seedling Stage-G124.csv - Growth Criteria Seedling Stage-G119.csv - Lipid Peroxidation.csv - Membrane Leakage.csv - Nitrogen (yield).csv - Nitrogen (seedling).csv - Peroxidase (seedling).csv - Phosphorous (seedling).csv - Phosphorous (yield).csv - Polyamines.csv - Potassium.csv - Proline Content (seedling stage).csv - Proline (yield).csv - Protein Nitrogen.csv - Sodium-Potassium.csv - Succulence – Root.csv - Succulence – Shoot.csv - Total Soluble Carbohydrates (Preflowering).csv - Total Soluble Carbohydrates (seedling).csv - Total Soluble Carbohydrates (yield).csv Data are available under the terms of the Creative Commons Zero “No rights reserved” data waiver (CC0 1.0 Public domain dedication).
