# Supplementary material for: Effect of polyamine precursors and antioxidants on growth and metabolism of salt-stressed barley
Source: F1000Res. 2024 Jan 18;12:262. Originally published 2023 Mar 10. [Version 2] doi: 10.12688/f1000research.130979.2 (PMC11522708; doi:10.12688/f1000research.130979.2)
Supplement: Peer review history

# Reviewer report: Reviewer response for version 2

**DOI:** 10.5256/f1000research.160909.r325303

**Published:** 2024-10-29

**Authors:** Mona F A Dawood

**Affiliations:** 1 Botany and Microbiology Department, Faculty of Science, Assiut University, Asyut, Assiut Governorate, Egypt

Title, add the scientific name of barely

 Improve the aim of the work and make it in one paragraph. 

 The references should be updated, you can take benefits from the following links

 Abdelrhim A, et al., 2024 (Ref 1)

 Dawood MFA, et al., 2023 (Ref 2)

 Sheteiwy MS, et al., 2022 (Ref 3)

 Ragaey MM, et al., 2022 (Ref 4)

 For paragraphs of ROS, glutathione, ascorbic acid, remove them to be before you writing about polyamines. 

 After polyamines, write on the exogenous application of ascorbic acid and glutathione.

 In materials, for the treatments, combine them in one paragraph and add a graphical chart represent your work. 

 Why you use the concentration of 0.1 mM NaCl salinity? Importantly, the concentration of Sodium chloride is too low, many studies indicated concentrations from 50 mM to 200 or 300 mM.     

  How the plants irrigated? How many times you add the solution?

 Swap (amino acid mixture) with polyamines precursors. 

 Add more  details on determination of biogenic amines by thin-layer chromatography (TLC).

 For statistics, use  ANOVA test to illustrate the statistical differences between treatments, 

 In results,

 remove a and b from writing the results. Then, you should add percent of changes to make it more clear. 

 After adding ANOVA, write on the statistical differences between treatments. 

 Some Figures did not have standard error, please add to them.

 For result, choose using figures or tables for representing traits measured. I prefer using figures. 

 Discussion

 1. Stay focused on the main findings of the study and avoid introducing too much background information.

 2. Discuss the results in the context of existing literature and explain the mechanisms behind the observed effects.

 3. Acknowledge the limitations of the study and suggest future research directions.

 Conclusion

 1. Briefly summarize the main findings of the study in a clear and concise way.

 2. Highlight the practical implications of the findings, especially for pest management and sustainable agriculture.

 Do not simply repeat information already mentioned in the Results or Discussion sections.

 Language

 1. Some sentences can be shortened or rephrased to be more concise.

 2. Use active voice whenever possible.

 3. Proofread carefully for typos and grammatical errors.

 4. Use consistent formatting throughout the sections.

 By addressing these areas for improvement, you can strengthen the "Results, Discussion and Conclusion" sections of your manuscript and make it more impactful for readers.

 I hope this feedback is helpful!

Is the work clearly and accurately presented and does it cite the current literature?

Yes

If applicable, is the statistical analysis and its interpretation appropriate?

Partly

Are all the source data underlying the results available to ensure full reproducibility?

Yes

Is the study design appropriate and is the work technically sound?

Partly

Are the conclusions drawn adequately supported by the results?

Partly

Are sufficient details of methods and analysis provided to allow replication by others?

Partly

Reviewer Expertise:

Stress Plant Physiology

I confirm that I have read this submission and believe that I have an appropriate level of expertise to confirm that it is of an acceptable scientific standard, however I have significant reservations, as outlined above.

## References

[1] : The role of Trichoderma koningii and Trichoderma harzianum in mitigating the combined stresses motivated by Sclerotiniasclerotiorum and salinity in common bean (Phaseolusvulgaris). *Plant Stress* .2024;11: 10.1016/j.stress.2024.100370 10.1016/j.stress.2024.100370

[2] : Role of Acetic Acid and Nitric Oxide against Salinity and Lithium Stress in Canola (Brassica napus L.). *Plants (Basel)* .2023;13(1) : 10.3390/plants13010051 10.3390/plants13010051 38202358 PMC10781170

[3] : Association of jasmonic acid priming with multiple defense mechanisms in wheat plants under high salt stress. *Front Plant Sci* .2022;13: 10.3389/fpls.2022.886862 886862 10.3389/fpls.2022.886862 36061773 PMC9429808

[4] : Role of Signaling Molecules Sodium Nitroprusside and Arginine in Alleviating Salt-Induced Oxidative Stress in Wheat. *Plants (Basel)* .2022;11(14) : 10.3390/plants11141786 10.3390/plants11141786 35890419 PMC9319962

# Reviewer report: Reviewer response for version 2

**DOI:** 10.5256/f1000research.160909.r238894

**Published:** 2024-02-21

**Authors:** Chandra Shekhar Seth

**Affiliations:** 1 Department of Botany, University of Delhi, New Delhi, Delhi, India

The manuscript is revised satisfactorily. I have no more comments.

Is the work clearly and accurately presented and does it cite the current literature?

Yes

If applicable, is the statistical analysis and its interpretation appropriate?

I cannot comment. A qualified statistician is required.

Are all the source data underlying the results available to ensure full reproducibility?

No source data required

Is the study design appropriate and is the work technically sound?

Yes

Are the conclusions drawn adequately supported by the results?

Yes

Are sufficient details of methods and analysis provided to allow replication by others?

Yes

Reviewer Expertise:

Stress Physiology

I confirm that I have read this submission and believe that I have an appropriate level of expertise to confirm that it is of an acceptable scientific standard.

# Reviewer report: Reviewer response for version 1

**DOI:** 10.5256/f1000research.143781.r221298

**Published:** 2023-11-30

**Authors:** Chandra Shekhar Seth

**Affiliations:** 1 Department of Botany, University of Delhi, New Delhi, Delhi, India

**Reviewer’s comments to Authors- **

1. While there is value in compilation the paper needs a revision due to several minor mistakes occurring throughout the manuscript. The written language needs to be improved.

**Abstract:**

1. The section describing the method can be crisper while elaborating the result and conclusion

**Introduction:**

1. The objectives of this study are missing in the introduction section.
2. “Because of the negative effect of salt stress on crop productivity, crop productivity loss might exceed 60% of crop yield globally”. Rephrase the sentence, repetition of crop productivity.
3. “Furthermore, salinity has impacted plant growth and development by increasing salt content, particularly ionic chloride cl and sodium Na+”. Correct symbol Chloride (Cl).
4. “Plants respond to salt stress by producing biochemical compounds and activating molecular systems at the cellular and plant levels (Li *et al*., 2019). Most cultivated plant species are highly sensitive and either die or display reduced productivity after exposure to salt stress for long periods.” Instead of generalized plant impact you should focus on barley salinity stress.

**Materials and Methods:**

1. “Grains were presoaked for 24 hrs in distilled water or amino acid mixture (Arginine, methionine and ornithine) 1mM for each or 0.1 mM for glutathione or 1 mM for ascorbic acid.” You mean using the amino acid mixture, glutathione, ascorbic acid alone separately. Rephrase this statement accordingly for clarification.
2. “One-week old seedlings were grown under the combined effect of 0.1 mM glutathione plus salt”. It means both were applied in the soil or firstly treated with glutathione and grown after in salt applied soil? Please clarify.
3. For pigment estimation optical densities are taken of acetone extract on the same day otherwise there is possible changes in the reading. Please confirm the methodology and clarify.
4. Estimate the initial fluorescence (F0). Initial fluorescence is represented as F _o_ not as F0. Check and correct.
5. The lipid peroxidation was measured in the seedling stage by the amount of malonyl dialdehyde (MAD). Malonyl dialdehyde is abbreviated as MDA not as MAD. Correct at all places.

**References:**

1. Maintain uniformity in referencing. Try to add DOI for each reference.
2. The different sections of the manuscript are poorly cited with references and required updating and validation with previous studies. The relevant papers listed below ^1^^-^^5^ may be considered to enhance the scientific quality of the manuscript significantly.

**Results and discussion:**

1. Write about the statistical analysis, how it is done using what procedure etc. Show the significant differences using alphabets.
2. Considering the result obtained the discussion is concise. Enrich it.

**Conclusion:**

1. The conclusion section of a review should be in accordance to the content.
2. Conclusion section failed to enlighten the spirit of the finding. Revise it precisely.
3. Conclusion section must also include the future perspectives of this study which is lacking in the manuscript.

Is the work clearly and accurately presented and does it cite the current literature?

Yes

If applicable, is the statistical analysis and its interpretation appropriate?

I cannot comment. A qualified statistician is required.

Are all the source data underlying the results available to ensure full reproducibility?

No source data required

Is the study design appropriate and is the work technically sound?

Yes

Are the conclusions drawn adequately supported by the results?

Yes

Are sufficient details of methods and analysis provided to allow replication by others?

Yes

Reviewer Expertise:

Stress Physiology

I confirm that I have read this submission and believe that I have an appropriate level of expertise to state that I do not consider it to be of an acceptable scientific standard, for reasons outlined above.

## References

[1] : Titanium dioxide nanoparticles potentially regulate the mechanism(s) for photosynthetic attributes, genotoxicity, antioxidants defense machinery, and phytochelatins synthesis in relation to hexavalent chromium toxicity in Helianthus annuus L. *J Hazard Mater* .2023;454: 10.1016/j.jhazmat.2023.131418 131418 10.1016/j.jhazmat.2023.131418 37104951

[2] : Interactive role of exogenous 24 Epibrassinolide and endogenous NO in Brassica juncea L. under salinity stress: Evidence for NR-dependent NO biosynthesis. *Nitric Oxide* .2020;97: 10.1016/j.niox.2020.01.014 33-47 10.1016/j.niox.2020.01.014 32045686

[3] : Review on nitric oxide at the forefront of rapid systemic signaling in mitigation of salinity stress in plants: Crosstalk with calcium and hydrogen peroxide. *Plant Sci* .2023;336: 10.1016/j.plantsci.2023.111835 111835 10.1016/j.plantsci.2023.111835 37611833

[4] : 24-Epibrassinolide Regulates Functional Components of Nitric Oxide Signalling and Antioxidant Defense Pathways to Alleviate Salinity Stress in Brassica juncea L. cv. Varuna. *Journal of Plant Growth Regulation* .2023;42(7) : 10.1007/s00344-022-10884-y 4207-4222 10.1007/s00344-022-10884-y

[5] : Nitric oxide mediated regulation of ascorbate-glutathione pathway alleviates mitotic aberrations and DNA damage in Allium cepa L. under salinity stress. *Int J Phytoremediation* .2023;25(4) : 10.1080/15226514.2022.2086215 403-414 10.1080/15226514.2022.2086215 35758213

# Response

**Published:** 2024-01-01

**Authors:** EMAN ELDAKKAK

**Affiliations:** Southern University at Baton Rouge, USA

1. A comprehensive modification was made to the abstract, adding new information and improving language according to the reviewer's suggestions.

 2. The introduction was strengthened by adding the objectives with a more extensive range of references to support the context.

 3. I rephrased and revised the sentences as per the reviewer's suggestion.

 4. In the methodology section, the initial part was reworded to enhance clarity regarding the treatments and align the pigment approach more cohesively. Furthermore, a detailed overview of the statistical analysis process was provided. 

  5. The discussion section was enhanced by improvements that provided further details regarding the findings and perspectives from earlier studies.

 6. The conclusion section was rephrased, incorporating perspectives for future research directions to provide a more forward-looking conclusion.

 7. More citations were incorporated. Additionally, more keywords were added and categorized alphabetically for better search engine performance.

# Reviewer report: Reviewer response for version 1

**DOI:** 10.5256/f1000research.143781.r186050

**Published:** 2023-08-30

**Authors:** Nasim Ahmad Yasin

**Affiliations:** 1 University of the Punjab, Lahore, Punjab, Pakistan

Authors of the manuscript “Effect of polyamine precursors and antioxidants on growth and metabolism of salt-stressed barley” observed that salinity stress resulted in the reduction of all growth parameters, pigment contents, photosynthetic efficiency, total soluble carbohydrates and grain weight in the seedlings, pre-flowering, and grain yield stages. On the other hand, salinity enhanced the activities of the antioxidant enzymes, catalase, and peroxidase, and increased the proline content of the three growth stages. The addition of glutathione, ascorbic acid, or amino acid mixture led to the enhancement of the defense mechanism of barley against salinity stress which was reflected in all previous parameters.

 This study is interesting. However, authors need to discuss the following issues:

1. Please add some data of your findings in the abstract section. Additionally, write benefits of polyamines precursors in treated plants.
2. What is the novelty of this study?
3. What was the hypothesis of this study?
4. Please rewrite following statements:
  “The pots were divided into five categories: the grains were presoaked for 24 hours in distilled water, an amino acid solution (Arginine, Methionine, and Ornithine) (1 mM each), glutathione, or ascorbic acid (0.1 mM for each), seedlings were cultivated in 0.1 mM sodium chloride alone, seedlings were cultivated using a combination of salt and glutathione, seedlings were grown using a combination of salt and ascorbic acid, seedlings were cultivated using a combination of salt and amino acid mixture”.
  “The addition of glutathione, ascorbic acid, or amino acid mixture alleviated the harmful effect of sodium chloride and improved barley's defense mechanism against salt stress”.
  “It has become widely known that reactive oxygen species (ROS) are to responsible for a diverse variety of stress-induced damage to macromolecules and, ultimately, cellular structure (Sairam et al., 2002)”.
  “In view of the lack of knowledge regarding the role of polyamines in salt tolerance of grain crops, it is attempted in the present study examines the effect of polyamine precursors; arginine, methionine, and ornithine, besides the effect of some antioxidants as glutathione and ascorbate on growth, metabolism, and productivity of two barley cultivars, different in salt tolerance, when subjected to salt stress”.
  “Giza 124 was chosen as the most tolerant cultivar and Giza 119 as the most sensitive one with 100 mM NaCl to be used in the present study”.
5. Write keywords alphabetically. Don’t use that word as a key word which is part of the title.
6. Why the cultivars Giza117, Giza119, Giza 121, Giza 123, Giza 124, Giza 125, Giza 126 and Giza 2000 were used for the current study?
7. Please clearly describe each treatment.
8. Why seedlings exposed to salt stress after a week?
9. How antioxidant solution was applied?
10. What do you mean by clay sandy soil (2:1)?
11. Describe the physicochemical characteristics of the soil used.
12. On what basis chemicals concentrations were selected to observe the stress alleviation response?
13. Please discuss the mechanisms through which salinity or antioxidants application resulted physiological/ biochemical or morphological modulations in treated plants.
14. Do you feel that this study provides an economical and eco-friendly solution for mitigation of salt stress?
15. What are future perspectives of this study?
16. Replace old references with latest ones.

Is the work clearly and accurately presented and does it cite the current literature?

Partly

If applicable, is the statistical analysis and its interpretation appropriate?

Partly

Are all the source data underlying the results available to ensure full reproducibility?

Yes

Is the study design appropriate and is the work technically sound?

Partly

Are the conclusions drawn adequately supported by the results?

Yes

Are sufficient details of methods and analysis provided to allow replication by others?

Partly

Reviewer Expertise:

NA

I confirm that I have read this submission and believe that I have an appropriate level of expertise to confirm that it is of an acceptable scientific standard, however I have significant reservations, as outlined above.

# Response

**Published:** 2024-01-01

**Authors:** EMAN ELDAKKAK

**Affiliations:** Southern University at Baton Rouge, USA

1. Why the cultivars Giza117, Giza119, Giza 121, Giza 123, Giza 124, Giza 125, Giza 126, and Giza 2000 were used for the current study?

 These cultivars were used at the beginning of the study as a first step to determine their tolerance and sensitivity. The decision to choose only two species was made from the beginning, prompting us to assess various cultivars to inform our final selection.

 2. What is the novelty of this study? 

 The novelty of this study lies in its investigation into the impact of polyamine precursors and antioxidants on the growth, metabolism, and productivity of two barley cultivars with varying salt tolerance when exposed to salt stress. Specifically, the study explores the effects of presoaking seeds in different solutions, including amino acid mixtures (Arginine, Methionine, and Ornithine), glutathione, and ascorbic acid, in the context of salt-tolerant (G124) and salt-sensitive (G119) barley cultivars.

 3. Why are seedlings exposed to salt stress after a week?

 Exposing the seedlings to salt stress after a week serves a specific purpose in this experiment. The one-week duration allows the seedlings to establish and develop to a certain extent before subjecting them to salt stress. This initial growth period enables the seedlings to form a stable root system and develop basic physiological structures.

 4. What was the hypothesis of this study?

 Hypothesis 1 states that the salt-tolerant barley cultivar (G124) will exhibit better growth parameters, higher photosynthetic pigments, increased total soluble carbohydrates, greater weight, and a higher number of grains compared to the salt-sensitive cultivar (G119) under salt stress conditions.

 Hypothesis 2 states that activities of antioxidant enzymes (catalase and peroxidase), proline content, malondialdehyde levels, and membrane leakage will be altered in response to salt stress, and the addition of polyamine precursors and antioxidants will mitigate these changes in both barley cultivars.

 Hypothesis 3 states that the combination of polyamine precursors and antioxidants will act cooperatively in alleviating the harmful impact of salt stress on barley cultivars, leading to enhanced defense mechanisms and improved plant growth.

 5. How antioxidant solution was applied?

 The antioxidant solution (glutathione or ascorbic acid) has been dissolved in water and applied directly to the soil surrounding the plant roots. This method ensures that the antioxidants are taken up by the plant roots and transported throughout the entire plant.

 6. What do you mean by clay sandy soil (2:1)?

 The term "clay sandy soil (2:1)" refers to a soil mixture composed of clay and sand in a specific ratio. In this case, the ratio is 2 parts of clay to 1 part of sand. This ratio indicates the proportion of the two soil components in the mixture.

 7. Describe the physicochemical characteristics of the soil used.

 Clay soil is characterized by its fine particles and tends to hold water and nutrients well. However, it can have poor drainage and may compact easily. It has a higher cation exchange capacity (CEC), which means it can hold more nutrients for plant uptake. On the other hand, sandy soil has larger particles and generally provides good drainage. However, it may not retain water and nutrients as effectively as clay. Sandy soils often have a lower CEC.

 8. On what basis chemical concentrations were selected to observe the stress alleviation response?

 We experimented with several salt concentrations to find the most suitable level. For other treatments, we relied on insights from prior research studies and reports in comparable experiments.  Additionally, the identification of a salt-tolerant cultivar (Giza 124) highlights the environmentally beneficial practice of cultivating naturally resistant crops.

 9. Do you feel that this study provides an economical and eco-friendly solution for the mitigation of salt stress?

 The study will have a significant economic impact, particularly if the recommended treatments work effectively in real agricultural settings. These treatments might be an affordable choice that benefits farmers financially by raising crop yields and improving crop quality.

 10. What are the future perspectives of this study?

 Future perspectives could include long-term evaluations of the sustainability and practical applicability of the suggested treatments and the salt-tolerant cultivar (Giza 124). Practical insights could be obtained through field trials conducted in various environmental conditions and integrated into precision agriculture procedures (GPS, sensors, etc..). In addition, research in molecular and genetics may reveal underlying systems that aid in the creation of genetically engineered crops.
